# Human resources for antimicrobial stewardship: a systematic review and exploratory meta-analysis

**DOI:** 10.1080/20523211.2026.2670702

**Published:** 2026-05-27

**Authors:** Somying Pumtong, Luerat Anuratpanich, Sermsiri Sangroongruangsri, Saranya Khunjan, Wacharapol Saengsiwaritt, Ukrit Sittiboot, Anh Phuong Ngoc Ta, Kumthorn Malathum, Nithima Sumpradit, Saowalak Turongkaravee

**Affiliations:** a Social, Economic and Administrative Pharmacy Division, Faculty of Pharmacy, Mahidol University, Bangkok, Thailand; b Social, Economic and Administrative Pharmacy Program, Faculty of Pharmacy, Mahidol University, Bangkok, Thailand; c Program Department of Biochemistry, Faculty of Pharmacy, Mahidol University, Bangkok, Thailand; d Faculty of Medicine, Ramathibodi Hospital, Mahidol University, Bangkok, Thailand; e Food and Drug Administration, Ministry of Public Health, Nonthaburi, Thailand

**Keywords:** Antimicrobial stewardship, Human resources, Health workforce, Staffing patterns, Exploratory meta-analysis

## Abstract

**Background:**

Antimicrobial resistance (AMR) is a major global health threat, and antimicrobial stewardship programmes (ASPs) are a cornerstone strategy to optimise antimicrobial use. International guidelines emphasise multidisciplinary teams; however, evidence describing how stewardship functions are organised, and quantified across professional groups remains fragmented, particularly outside high-income settings. This review synthesised evidence on stewardship functions, activities, and reported full-time equivalent (FTE) staffing patterns.

**Methods:**

A systematic review was conducted in accordance with PRISMA guidelines. Medline, Scopus, Embase, CINAHL, CABI, and grey-literature sources were searched to November 2025. Studies reporting stewardship functions, activities, or FTE involvement among infectious disease (ID) physicians, ID clinical pharmacists, infection control nurses (ICNs), clinical microbiologists, and hospital epidemiologists were included. Narrative synthesis mapped functions across professional groups. Where data permitted, exploratory post-hoc quantitative synthesis of reported FTEs by hospital bed capacity was conducted using random-effects models. Study quality and bias were assessed.

**Results:**

Fifty studies met the inclusion criteria: 44 described stewardship functions and activities, and seven reported FTE data. Responsibilities were distributed across professional groups: ID physicians and ID clinical pharmacists were mainly involved in prescribing oversight and optimisation, ICNs in monitoring and education, clinical microbiologists in diagnostic stewardship, and hospital epidemiologists in surveillance and reporting. Reported FTE involvement varied widely across hospital sizes and contexts, reflecting heterogeneity in design, role definitions, and reporting practices. Exploratory pooled estimates suggested higher reported ID physicians and ID clinical pharmacists involvement with larger hospitals; however, this pattern was affected by heterogeneity and publication bias and should be interpreted as descriptive, not generalisable.

**Conclusions:**

Current evidence does not support definitive staffing benchmarks for antimicrobial stewardship. This review highlights variability in how stewardship functions are organised and resourced. Future research should improve comparability through consistent reporting of stewardship activities, workforce roles, and institutional context.

## 1. Background

Antimicrobial resistance (AMR) is a major global health threat that undermines decades of medical progress, causing over 1.27 million deaths annually and disproportionately affecting low- and middle-income countries (LMICs), where access to effective antimicrobials is often limited (Murray et al., 2022; WHO, 2023). Its clinical and economic impact, including prolonged hospitalisation, higher mortality, and increased healthcare costs, highlights the need for coordinated interventions (Barlam et al., 2016).

Antimicrobial stewardship programmes (ASPs) are a key strategy to optimise antimicrobial use. ASPs are defined as ‘coordinated interventions designed to improve and measure the appropriate use of antibiotic agents by promoting the selection of the optimal antibiotic drug regimen, including dosing, duration of therapy, and route of administration' (Barlam et al., 2016). However, ASP implementation varies widely due to differences in infrastructure, resources, and workforce capacity. While high-income countries (HICs) have established structured stewardship teams, LMICs face persistent challenges such as limited staffing, inadequate training, and competing clinical demands (Barlam et al., 2016; Brink et al., 2016). Effective ASP implementation depends on multidisciplinary collaboration. International guidelines from SHEA, IDSA, and PIDS identify infectious disease (ID) physicians, ID clinical pharmacists, infection control nurses (ICNs), clinical microbiologists, and hospital epidemiologists as core ASP team members (Barlam et al., 2016). However, quantitative evidence describing how stewardship activities are resourced across these professional groups remains limited, particularly outside high-income settings. Existing studies are heavily concentrated in HICs and primarily describe heterogeneous staffing patterns rather than providing generalisable workforce benchmarks, creating uncertainty about how stewardship work is organised and resourced across diverse health systems (Greene et al., 2020; WHO, 2019). As a result, there is a need for a clearer understanding of how ASPs activities are currently resourced in practice, rather than an assumption that uniform staffing models can be applied across settings. This is especially important in LMIC contexts, where stewardship responsibilities are often integrated into existing clinical roles, involve task-shifting, or depend on flexible team structures. Synthesising available evidence on stewardship workforce roles and reported staffing patterns may therefore help inform institutional and policy-level discussions on context-sensitive workforce planning, while also clarifying the limitations of the current evidence base. Accordingly, this study systematically reviews the literature on antimicrobial stewardship workforce roles by synthesising evidence on core functions, activities, and reported full-time equivalent (FTE) staffing patterns of key healthcare professionals involved in hospital-based ASPs across different hospital capacities and income settings. The specific research questions addressed are: (1) How are antimicrobial stewardship functions and activities distributed across key healthcare professional groups in hospital-based ASPs? and (2) What patterns of reported FTE involvement have been described, and how do these vary by hospital size and health system context? This review aims to characterise how stewardship functions are distributed across professional groups and to examine reported FTE staffing data in order to identify patterns, variability, and critical evidence gaps across different hospital sizes and health system contexts.

## 2. Methods

This review was conducted following the guidelines of the Preferred Reporting Items for Systematic Reviews and Meta-Analysis (PRISMA) (Moher et al., 2009). This review protocol was registered in the PROSPERO database (CRD42023399688; https://www.crd.york.ac.uk/PROSPERO/view/CRD42023399688).

### 2.1. Identification of studies

We systematically searched Medline (via PubMed), Scopus, Embase, CINAHL, CABI and a targeted grey-literature search of key public health and stewardship websites (i.e. WHO, CDC, ECDC) through November 1, 2025. The search terms were constructed based on the PICOS domains (patient, intervention, comparison, outcome, and study type). The search terms were comprised of two domains: (1) antimicrobial stewardship intervention, and (2) workforce-related outcomes, including professional roles, stewardship activities, and full-time equivalent (FTE) staffing. The search focused on five healthcare professional groups commonly involved in antimicrobial stewardship: (1) infectious disease (ID) physicians, (2) ID clinical pharmacists, (3) infection control nurses (ICNs), (4) clinical microbiologists^1^, and (5) hospital epidemiologists. Full search strategies are provided in Supplemental Table S1. Searches were updated every three months, and reference lists of included studies and relevant grey literature were manually screened to identify additional eligible records.

### 2.2. Selection of studies

Two researchers (SK and WS) independently selected studies and were responsible for screening titles and abstracts. If they met the eligibility criteria, the full articles were retrieved. The studies were included if they met all the following criteria. First, studies that examined the human resources related to antimicrobial stewardship among five healthcare professionals, including (1) ID physicians, (2) ID clinical pharmacists, (3) ICNs, (4) clinical microbiologists, and (5) hospital epidemiologists. Second, they were studied and considered either (1) antimicrobial stewardship core functions and activities or (2) full-time equivalent staff (FTE) among those five healthcare professionals. Studies were excluded if they were not published in English and could not access the full-text article. Any disagreements were resolved through consensus with the third author (ST).

### 2.3. Data extraction

Data were extracted independently by two reviewers (SP and LA) using a piloted extraction form on a sample of five studies to ensure consistency. Extracted data included study characteristics (author, year, country, setting, and healthcare professional groups represented) and stewardship-related findings. Extracted outcomes were categorised into two components: (1) core antimicrobial stewardship functions and activities, and (2) reported FTE staffing data for stewardship-related roles, including measures of variance where available. Stewardship activities were defined a priori according to six functional domains: prescribing oversight and policy leadership; antimicrobial optimisation; monitoring, audit, and feedback; decision-support and diagnostics integration; communication and multidisciplinary collaboration; and education and capacity building.

### 2.4. Data analysis and synthesis

The narrative synthesis focused on important contextual factors of the available literature, including the country's analyses, healthcare professionals represented, types of settings, functions/roles, and activities in the ASPs, and the number of FTEs among five healthcare professionals. The country-level data was aggregated regionally and into income brackets based on the 2021 revised categorisations of the World Bank. The country income levels were classified according to the World Bank income classifications: lower-middle-income economies are those with a GNI per capita between $1,136 and $4,465; upper-middle-income economies are those with a GNI per capita between $4,466 and $13,845; high-income economies are those with a GNI per capita of $13,846 or more (The World Bank, 2024).

The quantitative synthesis of FTE data was not pre-specified in the original PROSPERO protocol. Following data extraction, a subset of studies reporting comparable FTE measures was identified, and an exploratory, post-hoc synthesis was conducted after data inspection. Accordingly, the pooled estimates should be interpreted as descriptive and hypothesis-generating representations of reported practice, rather than as normative benchmarks for workforce planning. This analysis was intended to characterise patterns, variability, and dispersion in reported stewardship staffing, rather than to estimate optimal, recommended, or generalisable workforce levels. For each professional group, mean FTEs values were pooled by hospital bed capacity where at least two studies reported comparable data. Although institutional characteristics such as teaching status, academic affiliation, and urban–rural location plausibly influence stewardship workload, these variables were inconsistently reported and rarely linked directly to FTE estimates across studies. As a result, formal subgroup analyses or meta-regression to explore sources of heterogeneity were not methodologically feasible.

For the narrative synthesis, stewardship activities were independently coded by two reviewers (SP and LA) using this predefined framework. Prior to full coding, the reviewers jointly piloted the coding approach on a subset of included studies to ensure consistent interpretation of activity definitions. Coding discrepancies were resolved through consensus discussion, and where agreement could not be reached, adjudication was provided by a third reviewer (ST). A formal inter-rater agreement statistic was not calculated for the full coding phase, as discrepancies were resolved through iterative discussion and adjudication within a predefined coding framework. This approach is consistent with qualitative and framework-based narrative synthesis methods, where interpretive consensus is prioritised over statistical agreement.

### 2.5. Statistical analysis

For the exploratory quantitative synthesis, pooled mean FTE and their 95% confidence interval (CIs) were estimated separately for each healthcare professional and bed capacities if at least two studies were provided. Statistical heterogeneity was assessed using the *I²* statistic. Given the observed statistical heterogeneity (*I²* > 25% in most subgroups) and expected clinical heterogeneity across healthcare systems, a DerSimonian and Laird random-effects model was selected as the most appropriate analytic approach (DerSimonian & Laird, 1986). This decision was data-driven and reflects variability in institutional context, stewardship models, and reporting practices across countries rather than an assumption of underlying homogeneity. Moreover, the potential for publication bias was evaluated for subgroups with ten or more studies (N ≥ 10). Our evaluation included: (1) visual inspection of funnel plot asymmetry; (2) statistical testing using Egger's regression test (Minelli et al., 2005); and (3) contour-enhanced funnel plots to differentiate asymmetry caused by publication bias (i.e. missing studies in non-significant areas) from other factors (Egger et al., 1997). For subgroups with fewer than ten studies (N < 10), any bias assessment was considered inconclusive, as these tests are known to lack sufficient statistical power with a small number of studies.

For each professional group, mean FTEs values were pooled by hospital bed capacity where at least two studies reported comparable data. Hospital bed capacity was used as a pragmatic and consistently reported stratification variable across studies, enabling structured comparison in the absence of standardised institutional data. However, this variable captures only one dimension of institutional scale and does not account for structural differences across health systems, including variation in case mix, service complexity, laboratory infrastructure, availability of subspecialists, and stewardship programme maturity. Importantly, hospital bed-capacity categories were analysed as reported in the primary studies and were not standardised across health systems. This decision was made to preserve the original reporting context and to avoid introducing unsupported assumptions of comparability across structurally dissimilar institutions. As such, pooling across non-standardised bed categories should be understood as a methodological choice that exposes, rather than resolves, heterogeneity in the evidence base. Variation in pooled estimates should therefore be interpreted as reflecting underlying structural and contextual differences between studies, rather than statistically attributable effects. This synthesis was therefore undertaken to map variability, reporting patterns, and structural inconsistency in the available evidence, rather than to derive precise, comparable, or transferable estimates of antimicrobial stewardship workforce requirements. All analyses were performed using STATA software, version 18 using the built-in `metan` command for random-effects models. A *p*-value < 0.05 was considered statistically significant.

### 2.6. Quality assessment of evidence

The quality of the evidence was assessed across the various study designs using (1) JBI checklist for cross-sectional study (The Joanna Briggs Institute Critical Appraisal tools for use in JBI Systematic Reviews. Checklist for Analytical Cross Sectional Studies, 2017), (2) CASP checklist for qualitative study (*Critical Appraisal Skills Programme* (CASP), 2018), (3) AGREE-II checklist for guideline (APPRAISAL OF GUIDELINES FOR RESEARCH & EVALUATION II, 2017), and (4) SANRA checklist for narrative review (Baethge et al., 2019). Based on the JBI, studies scoring >70% were considered ‘high’ quality, 50-69% as ‘medium’ quality, and <50% as ‘low’ quality. A sensitivity analysis was performed by excluding studies rated as low quality and re-analysing the exploratory quantitative synthesis using only medium- and high-quality studies. For the other tools, an overall quality ranking was not derived, and their scores were used solely for descriptive purposes. Detailed of the quality score calculation are provided in Supplemental Table S9. Any disagreements encountered during the screening or data extraction phases were resolved through discussion with a third author (ST).

## 3. Results

### 3.1. Search results

**Figure 1.**
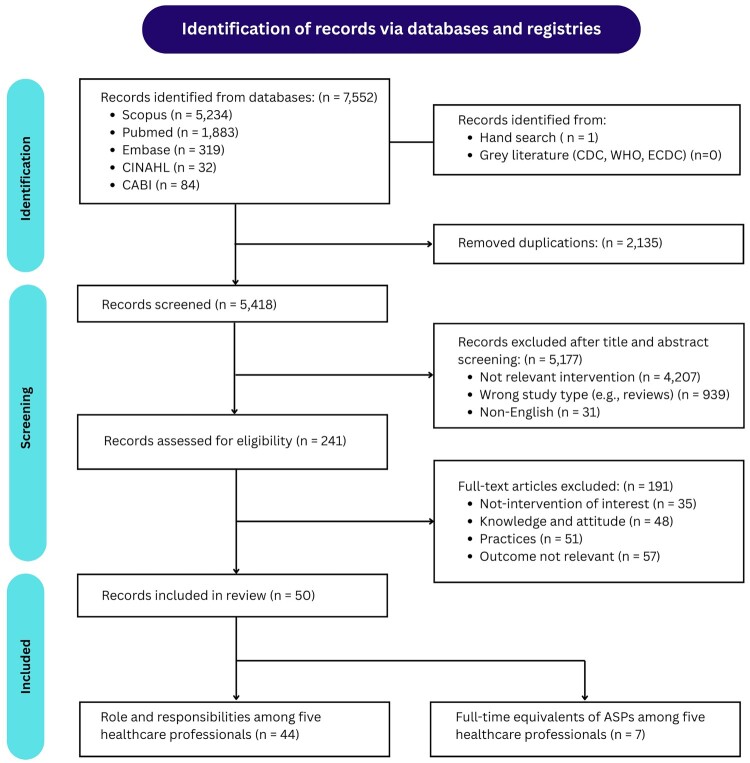
PRISMA flow diagram showing identification, screening, eligibility assessment, and inclusion of studies in the systematic review.

The database search identified 7,552 articles (5,234 from Scopus, 1,883 from PubMed, 319 from Embase, 84 from CABI, and 32 from CINAHL). After removing 2,135 duplicates, 5,418 titles and abstracts were screened, and 241 full-texts were reviewed. Following exclusions, 50 studies were included for data extraction, 44 focused on ASP roles (core functions) and activities, and 7 on FTEs among five healthcare professionals (Figure 1, Supplemental Table S2). The inter-rater reliability for the screening and quality appraisal was of high agreement between reviewers, with a Cohen’s kappa of 0.95 and 0.86 respectively.

### 3.2. ASP workforce across five healthcare professionals

The primary findings were divided into two parts: (1) core functions and activities of the five healthcare professions and (2) reported FTE staffing data across ASP healthcare professions

#### 3.2.1 Core functions and activities of the five healthcare professions

Between 2000 and 2024, 44 studies examined ASP activities, with nurses being the most studied professionals (n = 12), followed by pharmacists (n = 11), physicians (n = 4), clinical microbiologists (n = 4), and epidemiologists (n = 3). In addition, ten studies involved multi-sectoral healthcare teams. Most studies (67%) were conducted in HICs, primarily the United States (n = 18), followed by the United Kingdom (n = 4), France (n = 2), Australia and New Zealand, Japan and Canada. Three studies were from upper-middle-income countries (South Africa and China), and one from an upper-middle (Thailand) and a LMIC (Nigeria). Notably, no studies represented a LMIC in Southeast Asia.

Table 1 summarises stewardship activities across six core functional domains and five healthcare professional groups. ID physicians and clinical pharmacists were most frequently involved in prescribing oversight, antimicrobial optimisation, and audit/feedback activities. ICNs primarily contributed to monitoring, communication, and education functions, reflecting their close involvement in bedside practice. Clinical microbiologists were mainly engaged in diagnostic stewardship and surveillance activities, while hospital epidemiologists focused on surveillance, reporting, and coordination functions. Collectively, these findings illustrate complementary but function-specific roles across professional groups rather than uniform stewardship responsibilities (Figure 4). Detailed mappings are provided in Supplemental Tables S3–S7.

**Table 1.** Core functions and activities of ASP and healthcare professional involvement.

Core functions and activities	*P*	Rx	*E*	*M*	*N*
1. Prescribing oversight and policy leadership					
Developing clinical guidelines	/	/	/		
Implementing formulary restrictions	/	/	/		
Developing and maintaining antibiograms	/	/	/		
Documenting written policies and institutional commitments	/	/			
Utilising antimicrobial order forms	/	/			
2. Antimicrobial optimisation					
Implementing antimicrobial cycling strategies	/	/			
De-escalating antimicrobial therapy based on clinical and microbiological data	/	/			
Switching intravenous antibiotics to oral formulations	/	/			
Using combination therapy	/	/			
Optimising antimicrobial dosing	/	/			
Discontinuing antibiotics	/	/			
Implementing daily care bundles to optimise antibiotic use	/	/			/
3. Monitoring, audit, feedback, and surveillance					
Conducting ward-level/hospital-wide audits	/	/			
Conducting prospective audits with feedback	/	/			
Overseeing antimicrobial adherence and providing bedside consultation					/
Collecting and reporting normalised antimicrobial use data			/		
Reporting antimicrobial use and resistance information to clinical staff				/	
Conducting surveillance for emerging pathogens and AMR patterns				/	
4. Decision-support and diagnostics integration					
Using computerised order entry systems to support antimicrobial decision-making	/	/			
Providing point-of-care antimicrobial susceptibility reports	/	/			
Supporting clinical diagnostic tests for pathogen detection	/	/			
Ensuring availability of appropriate diagnostic testing				/	
5. Communication and multidisciplinary collaboration					
Collaborating with other healthcare professionals on infection prevention initiatives			/		
Conducting regular clinical quality meetings with multidisciplinary teams			/		/
Maintaining direct and indirect communication channels with prescribers			/	/	
Participating in regular multidisciplinary clinical quality meetings			/		/
6. Education and capacity building					
Providing education for medical staff	/	/	/	/	/
Providing education and consultation services for patients	/	/	/		/
Providing education for the public			/	/	/

*P* = ID physician, Rx = ID clinical pharmacist, *E* = hospital epidemiologist, *M* = clinical microbiologist, *N* = Infection control nurse,

HAIs = healthcare-associated infections.

#### 3.2.2 Reported FTE staffing patterns across ASP healthcare professions

This part demonstrated a quantitative synthesis of FTEs of ASPs among five healthcare professions. However, data was limited to studies that reported the staff FTEs for ASPs. Seven studies published between 2017 and 2022 were included. Notably, all studies were conducted in HICs, specifically the United States (n = 2) (Doernberg et al., 2018; Nhan et al., 2019), Japan (n = 3) (Maeda, Muraki, Kosaka, Yamada, Aoki, Kaku, Kawaguchi, et al., 2019a; Maeda, Muraki, Kosaka, Yamada, Aoki, Kaku, Seki, et al., 2019b; Umemura et al., 2022), the Netherlands (Kallen et al., 2018), and New Zealand (Gardiner et al., 2017). All studies were conducted in hospital settings across a range of bed capacities. The mean of FTEs was pooled in each healthcare profession as follows: ID physicians (n = 5), ID clinical pharmacists (n = 5), ICNs (n = 3), clinical microbiologists (n = 2), and hospital epidemiologists (n = 2); see detail in (Supplemental Table S8).

Across these studies, hospitals within similar bed-capacity categories represented heterogeneous institutional contexts, including differences in academic affiliation, stewardship programme maturity, specialist availability, and supporting infrastructure. For example, United States studies predominantly reflected academic or tertiary centres, whereas the Japanese nationwide survey included a broader range of hospitals, many without dedicated stewardship teams or on-site laboratory capacity. Dutch and New Zealand studies further reflected variation in organisational structure and stewardship leadership models.

(1) ID physicians

**Figure 2.**
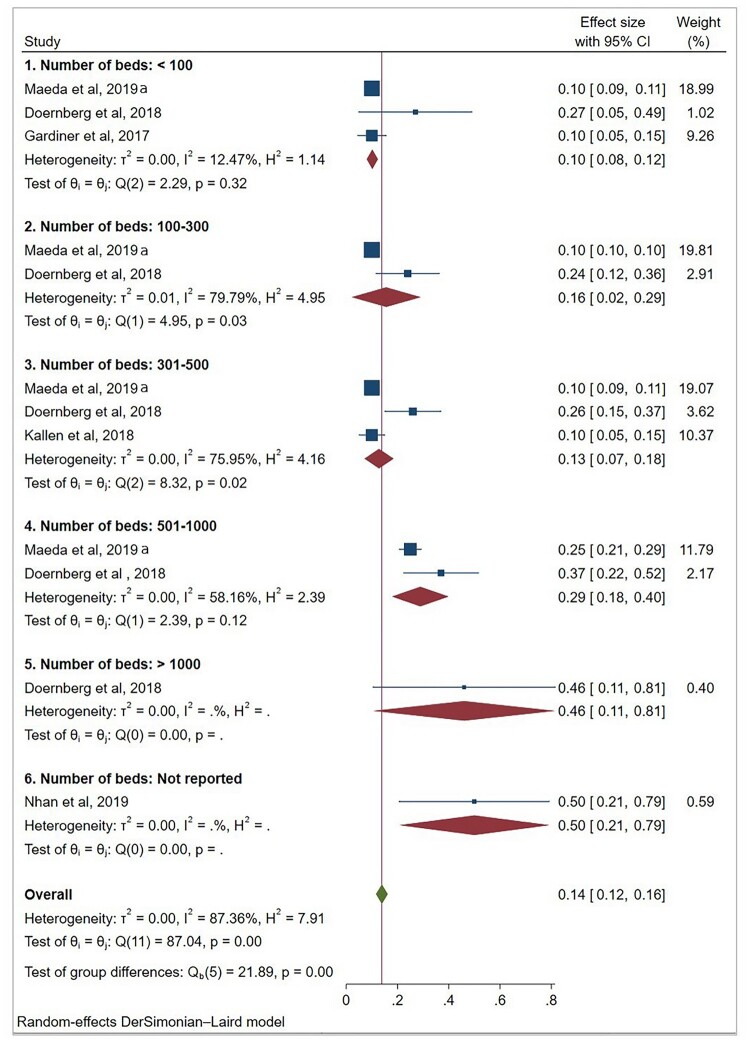
Forest plot of reported full-time equivalent staffing for infectious disease physicians by hospital bed-capacity category.

Five studies (12 sub-studies) reported FTE data for ID physicians. Pooled mean FTE values were 0.10 (95% CI 0.08–0.12) for hospitals with <100 beds, 0.16 (0.02–0.29) for 100–300 beds, 0.13 (0.07–0.18) for 301–500 beds, 0.29 (0.18–0.40) for 501–1,000 beds, and 0.46 (0.11–0.81) for >1,000 beds (Figure 2).

Substantial heterogeneity was observed across bed-capacity categories, particularly in the 100–300 and 301–500 bed groups. This variation reflects differences in institutional context and stewardship models that are not captured by hospital size alone. While higher FTE values were observed in larger hospitals, this pattern should be interpreted as a descriptive trend within heterogeneous data rather than as evidence of a consistent or proportional relationship between hospital size and staffing levels.

(2) ID clinical pharmacists

**Figure 3.**
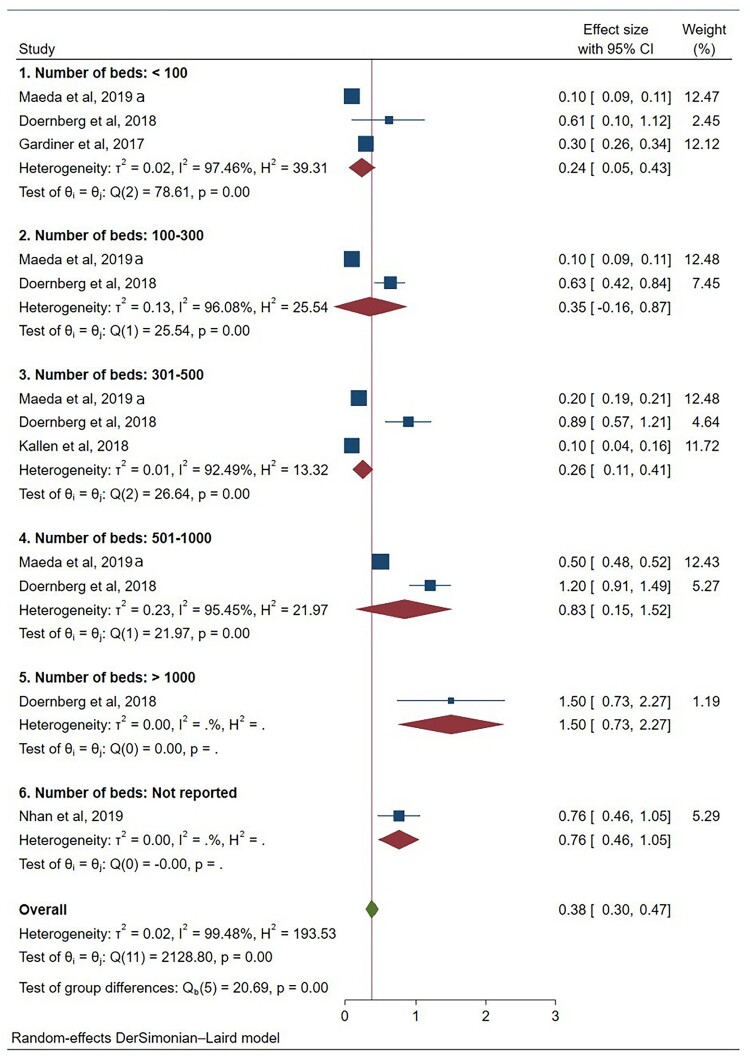
Forest plot of reported full-time equivalents staffing for infectious disease clinical pharmacists by hospital bed-capacity category.

Five studies (12 sub-studies) reported FTE data for ID clinical pharmacists. Pooled mean FTE values were 0.24 (95% CI 0.05–0.43) for <100 beds, 0.35 (0.16–0.87) for 100–300 beds, 0.26 (0.11–0.41) for 301–500 beds, 0.83 (0.15–1.52) for 501–1,000 beds, and 1.50 (0.73–2.27) for >1,000 beds (Figure 3). High heterogeneity was observed across all subgroups, indicating substantial variation in reported staffing models. While higher FTE values were reported in larger hospitals, these findings represent context-dependent patterns of reported practice rather than consistent scaling relationships. Variation likely reflects differences in stewardship programme structure, role definition, and reporting practices across settings.

(3) ICNs

**Figure 4.**
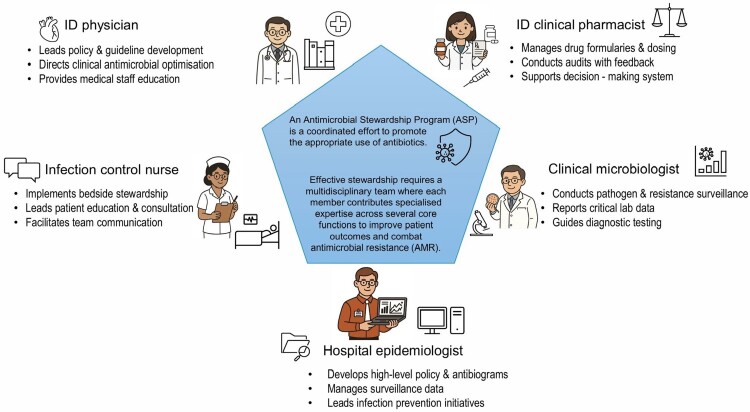
Conceptual framework of complementary multidisciplinary roles across core antimicrobial stewardship functions.

Three studies (with 6 sub-studies) assessed the FTEs of ASPs among infection control nurses. Due to the number of FTE nurses in each bed category, the capacity was only one to two studies, and the number of studies was insufficient for pooling. The results from the individual studies revealed that the number of nurses’ FTE in beds <100 and 100–300 beds was 0.10, bed 301–500 was 0.25-0.40, and in beds 501-1,000 beds was 0.20 (Supplemental Figure S3). The results suggested that the number of FTE nurses was a 2–4-fold increase in those exceeding 300 beds.

(4) Clinical microbiologists

Two studies (with 5 sub-studies) assessed the FTEs of ASPs among laboratory personnel (medical technologists, microbiologists, and laboratory technicians). There were not sufficient studies for pooling. The results from the individual studies revealed that the number of laboratory technicians’ FTE in beds 101–300 beds was 0.10, beds 301–500 was 0.10–0.20, and in beds 501–1,000 was 0.20 (Supplemental Figure S5). The results suggested that the number of FTE laboratory personnel doubled in those exceeding 300 beds.

(5) Hospital epidemiologists

Two studies assessed the FTEs of ASPs among epidemiologists in bed capacities of 301–500 beds and an undefined number of beds, respectively. The results indicated that the number of FTE epidemiologists in bed capacities 301–500 beds was 0.10 (Supplemental Figure S6).

It should be noted that these quantitative findings warrant cautious interpretation, as many contributing studies lacked clear definitions of stewardship FTE activities and did not adequately account for institutional characteristics, such as teaching status or service complexity, that are likely to influence reported staffing patterns.

### 3.3. Publication bias

For the ID physicians (N = 12) and ID clinical pharmacists (N = 12) subgroups, both of which met the minimum requirement of 10 studies for a reliable assessment, significant funnel plot asymmetry was observed (Egger's test, *P* < 0.001 for both groups). The corresponding contour-enhanced funnel plots further suggest that this asymmetry is probably caused by publication bias, indicating that studies are missing from the non-significant regions (*p* > 5%) of the plot (Supplemental Figures S7–S10). For the remaining subgroups, ICNs (N = 6), clinical microbiologists (N = 5), and hospital epidemiologists (N = 2), the number of included studies was below the recommended 10-study minimum. Since formal tests for publication bias (such as Egger's regression) lack enough statistical power and are unreliable with a small number of studies, the assessment for these groups was considered inconclusive (Supplemental Figures S11–S15).

### 3.4. Quality assessment of included studies

Most cross-sectional studies were rated as medium to low quality, primarily due to unclear definitions of stewardship FTE exposure and limited consideration of institutional or hospital-level confounders. As a result, reported FTE estimates may not be directly comparable across studies, further limiting the interpretability of pooled estimates beyond statistical heterogeneity alone. The qualitative study was of high quality, while guidelines scored highest on stakeholder involvement (83%) and scope (67%). Narrative reviews scored 4–12 out of 12 points, with ‘description of the literature search' scoring lowest and ‘statement of concrete aims' highest (Supplemental Table S9). For the quantitative synthesis, it was based solely on the seven cross-sectional studies that provided FTE data. For this group, we utilised their specific JBI quality ratings (high, medium, or low, as detailed in Supplemental Table S9(a)). Sensitivity analyses excluding low-quality studies did not materially change the pooled FTE estimates for physicians, pharmacists, or nurses, reflecting limited statistical power due to the small number of included studies. (Supplemental Table S10, Figures S1–S2, S4).

Most cross-sectional studies were rated as medium to low quality, primarily due to unclear definitions of stewardship FTE exposure and limited consideration of institutional or hospital-level confounders. As a result, reported FTE estimates may not be directly comparable across studies, further limiting the interpretability of pooled estimates beyond statistical heterogeneity alone. This quality-related comparability problem further constrains interpretation of pooled FTE estimates beyond statistical heterogeneity alone. For the narrative synthesis, we included all other studies, the remaining ten cross-sectional studies without FTE data, the narrative reviews, the guidelines, and qualitative studies. Since this was a heterogeneous group with different appraisal tools, we used their respective quality scores to provide descriptive context regarding the methodological strength of the evidence. In summary, across HIC settings, ID physicians and ID clinical pharmacists most consistently contributed to stewardship activities, with reported FTE involvement increasing in larger hospitals. However, hospitals within similar bed-capacity categories operated under substantially different institutional and health system contexts, which were inconsistently reported across studies. Consequently, high heterogeneity and limited contextual data constrain interpretation of pooled FTE estimates and preclude their application to specific institutional settings, underscoring the need for standardised reporting of hospital characteristics to support future comparative workforce analyses.

## 4. Discussion

This review consolidates how antimicrobial stewardship functions are distributed across five key healthcare professional groups. Rather than defining optimal staffing levels, our findings reveal both common functional patterns and substantial structural variations in how stewardship responsibilities are allocated across different institutional contexts and health systems.

Our exploratory quantitative synthesis of high-income settings suggests that reported stewardship staffing tends to increase with hospital size, particularly for ID physicians and ID pharmacists. However, substantial statistical heterogeneity and significant publication bias limit these findings to descriptive summaries rather than staffing requirements. The literature likely overrepresents well-resourced programmes, potentially creating upward-biased estimates. Evidence for nurses, microbiologists, and epidemiologists remains particularly limited (N < 10), highlighting critical gaps in workforce data. Importantly, this interpretation is further constrained by study quality; most contributing cross-sectional studies were rated as medium or low quality, often due to unclear definitions of stewardship activities and a lack of adjustment for institutional characteristics such as service complexity or teaching status. These limitations directly affect the comparability of reported FTE values and contribute to the observed variability across studies. The substantial heterogeneity observed across hospital bed-capacity categories should not be interpreted solely as a methodological limitation. Rather, it likely reflects underlying differences in institutional and health system characteristics not captured by bed size alone, including teaching status, academic affiliation, availability of subspecialists, stewardship programme maturity, and supporting infrastructure. As these characteristics were inconsistently reported and rarely linked to FTE estimates, their contribution to heterogeneity could not be formally examined, limiting the ability to explain observed variation or assess the context-specific applicability of reported staffing levels. In resource-constrained settings, antimicrobial stewardship is frequently implemented through task-shifting, role overlap, and reliance on generalist clinicians, with pharmacists or nurses undertaking responsibilities that would typically be performed by ID physicians in high-income contexts (Brink et al., 2016; Greene et al., 2020). Such adaptive models challenge conventional FTE-based accounting, as stewardship contributions are often embedded within broader clinical roles rather than allocated as discrete positions. Consequently, the application of staffing ratios derived from high-income settings risks both underestimating shared stewardship effort and overestimating workforce needs when individuals contribute across multiple functions. These observations are consistent with the WHO Practical Toolkit for Antimicrobial Stewardship in LMICs, which emphasises flexible, context-specific, and function-oriented workforce configurations rather than fixed staffing ratios. Within this framework, core stewardship functions, such as antimicrobial review, guideline implementation, surveillance, education, and audit and feedback, may be distributed across general physicians, pharmacists, nurses, and microbiologists depending on local capacity and service organisation. Importantly, this redistribution is not merely structural but functional: generalist clinicians and non-specialist staff may undertake protocol-driven activities such as antimicrobial review, guideline implementation, and routine audit and feedback, whereas more complex functions, including management of multidrug-resistant infections, interpretation of advanced microbiological data, and stewardship programme oversight, continue to rely on specialist input where available. In addition to task-shifting and structural flexibility, training and capacity-building models help operationalise this redistribution within existing workforces in resource-limited settings. The WHO toolkit highlights competency-based and function-oriented training approaches, whereby stewardship skills are developed through targeted in-service training, mentorship by regional or national experts, cascade training within institutions, and integration of stewardship competencies into routine clinical education for physicians, pharmacists, nurses, and microbiologists. These training-focused strategies enable generalist and non-specialist staff to undertake core stewardship activities without reliance on formal infectious disease training, thereby expanding stewardship capacity without proportional increases in specialist FTEs. Digital stewardship strategies further augment this model by extending specialist expertise beyond physical constraints. Evidence from electronic decision-support systems demonstrates improvements in prescribing accuracy, real-time audit and feedback, and reductions in inappropriate antimicrobial use (Yarahuan et al., 2023), while tele-stewardship models, including remote pharmacist support and virtual infectious disease consultation, have enabled facilities without on-site specialists to access stewardship expertise and maintain programme functionality (Chong et al., 2025). Taken together, these approaches illustrate that stewardship functions in LMICs are often redistributed across cadres, embedded within existing clinical roles, and selectively supported by remote or digital specialist input, rather than delivered through fixed specialist staffing structures alone. Viewed alongside the high-income country data synthesised in this review, these context-adapted models illustrate that variability in reported FTEs reflects not only differences in hospital size, but also fundamentally different organisational strategies for delivering antimicrobial stewardship. Recognising and synthesising these alternative workforce configurations is therefore essential for interpreting the existing evidence base and for guiding future research towards function-based, context-sensitive workforce planning. When compared with existing guideline recommendations, such as Canadian guidance suggesting one ID physician and three ID clinical pharmacists per 1,000 hospital beds (Morris et al., 2018), our pooled estimates are substantially lower (e.g. 0.29 physicians and 0.83 pharmacists for hospitals with 501–1,000 beds). This discrepancy reflects the fundamentally different purposes of the two evidence sources: guideline recommendations represent normative and aspirational targets within well-resourced systems, whereas our estimates summarise empirically reported staffing levels. Furthermore, differences in how stewardship activities are defined and operationalised, particularly the distinction between dedicated and embedded roles, limit direct comparability. This divergence, therefore, highlights the need to distinguish between aspirational standards and descriptive evidence when interpreting and transferring workforce guidance across a health system context.‏ Beyond staffing numbers, this review highlights that workforce constraints are deeply intertwined with broader system-level challenges. Limited managerial support, fragmented communication, inadequate national coordination, and weak information infrastructure continue to hinder ASP implementation, particularly in LMIC contexts (Alghamdi et al., 2019; Gardiner et al., 2017). These findings indicate that antimicrobial stewardship workforce limitations cannot be addressed in isolation but must be considered within the wider health system environment in which stewardship operates. This review has limitations that should be interpreted considering its contribution. First, the quantitative synthesis was based on a small number of HIC-based studies and was conducted post hoc, reinforcing its exploratory and descriptive nature rather than its suitability for workforce benchmarking. Second, restriction to English-language publications may have omitted pertinent experiences from LMICs. Third, heterogeneous reporting standards limited the comparability of available FTE data. At the same time, these limitations constitute key findings of the review, demonstrating the absence of standardised definitions, reporting practices, and analytic frameworks for antimicrobial stewardship workforce assessment. Future research should therefore prioritise context-adapted study designs, standardised reporting of stewardship activities, and analytic approaches that capture role sharing, task-shifting, and digital stewardship models, particularly in settings where the burden of antimicrobial resistance is most pronounced.

## 5. Conclusion

These FTE estimates represent descriptive summaries of reported staffing in well-resourced contexts and should not be interpreted as normative benchmarks for workforce planning. The findings demonstrate substantial variability in how stewardship activities are structured and resourced, reflecting differences in institutional roles, health system organisation, and reporting practices rather than consistent or comparable staffing patterns. The available evidence does not support the derivation of generalisable or context-independent workforce benchmarks. Instead, reported FTE values reflect heterogeneous and context-specific implementations of stewardship, shaped by variation in study design, role definition, and incomplete reporting of institutional characteristics. These patterns underscore that antimicrobial stewardship workforce configuration is inherently context-dependent and cannot be reduced to fixed ratios or universally applicable models.

Future research should focus on improving the interpretability and comparability of workforce evidence through more consistent reporting of stewardship activities, workforce roles, and institutional context, rather than on establishing prescriptive staffing targets.

## Supplementary Material

Supplemental Material

## Footnotes

1 ‘The term ‘clinical microbiologists’ refers to laboratory-based professionals involved in microbiological diagnostics and antimicrobial susceptibility testing, including clinical microbiologists, medical technologists, and laboratory technicians.'

## Data sharing statement

All data have been included in the manuscript. All data generated or analysed during this study are included within this published article and its Supplemental Material.
