# Supplementary material for: Human resources for antimicrobial stewardship: a systematic review and exploratory meta-analysis
Source: J Pharm Policy Pract. 2026 May 27;19(1):2670702. doi: 10.1080/20523211.2026.2670702 (PMC13218311; doi:10.1080/20523211.2026.2670702)
Supplement: Supplemental Material [file JPPP_A_2670702_SM9585.pdf]

Supplement data

Title: Human Resources for Antimicrobial Stewardship: A Systematic Review and Exploratory Meta-analysis

List of Table

Table S1 Search terms and strategies

Table S2 Characteristics of included studies (n = 50); a) Role and activities on ASPs among five healthcare professionals (N=44), b) The studies of FTE on antibiotic stewardship program (N=7)

Table S3 Core functions and activities in the ASPs of ID physicians (n = 13)

Table S4 Core functions and activities in the ASPs of ID clinical pharmacists (n = 21)

Table S5 Core functions and activities in the ASPs for ICNs (n = 13)

Table S6 Core functions and activities in the ASPs of clinical microbiologists (n = 8)

Table S7 Core functions and activities in the ASPs of hospital epidemiologists (n = 4)

Table S8 The number of FTEs of ASPs among five healthcare professionals

Table S9 Quality assessment of included studies

Table S10 Summary pooled for main and sensitivity analysis

Table S11 Assessment of publication bias

List of Figure

Figure S1 Sensitivity analysis by excluding low quality study. Forest plots for pooling the number of full-time equivalents of ASPs among infectious disease physicians

Figure S2 Sensitivity analysis by excluding low quality study. Forest plots for pooling the number of full-time equivalents of ASPs among infectious disease clinical pharmacists

Figure S3 Forest plots for pooling the number of full-time equivalents of ASPs among infection control nurses

Figure S4 Sensitivity analysis by excluding low quality study. Forest plots for pooling the number of full-time equivalents of ASPs among infection control nurses

Figure S5 Forest plots for pooling the number of full-time equivalents of ASPs among clinical microbiologists

Figure S6 Forest plots for pooling the number of full-time equivalents of ASPs among hospital epidemiologists

Figure S7 Funnel plots for infectious disease physicians

Figure S8 Contour enhanced funnel plots for infectious disease physicians

Figure S9 Funnel plots for infectious disease clinical pharmacists

Figure S10 Contour enhanced funnel plots for infectious disease clinical pharmacists

Figure S11 Funnel plots for infection control nurses

Figure S12 Contour enhanced funnel plots for infection control nurses

Figure S13 Funnel plots for clinical microbiologists

Figure S14 Contour enhanced funnel plots for clinical microbiologists

Figure S15 Funnel plots for hospital epidemiologists

Table S1 Search terms and strategies

a) Medline via Pubmed database

Search term				
**Antibiotics/antimicrobial resistance management**		**Human resource**		**Occupation**
(anti-bacterial*OR antibacterial* OR anti-mycobacterial* OR antimycobacterial* OR anti-fung* OR antifung* OR anti-biotic* OR antibiotic* OR anti-microbial* OR antimicrobial* OR anti-infective*) OR [MeSH] (anti-infective agents) AND (stewardship*OR prevent* OR management OR control) OR [Mesh] ("Antimicrobial Stewardship")	AND	(Health OR healthcare OR health care) AND [Title/Abstract] (employee*OR labor* OR labour* OR staff* OR team* OR worker* OR workforce* OR profession* OR personnel*) OR (Manpower) OR [Mesh] (Human resource)	AND	(doctor*OR physician* OR practitioner* OR clinician* OR pharmacist* OR nurse* OR epidemiologist*) OR (medical OR microbiology OR laboratory) OR [Mesh] (Infection control practitioners)

b) Scopus database

Search term				
**Antibiotics/antimicrobial resistance management**		**Human resource**		**Occupation**
TITLE-ABS-KEY (anti-bacterial* OR antibacterial* OR anti-mycobacterial* OR antimycobacterial* OR anti-fung* OR antifung* OR anti-biotic* OR antibiotic* OR anti-microbial* OR antimicrobial* OR anti-infective*) AND TITLE-ABS-KEY (stewardship* OR prevent* OR management OR control OR TITLE-ABS-KEY ("antimicrobial stewardship")	AND	TITLE-ABS-KEY (health OR healthcare OR "health care") AND TITLE-ABS-KEY (employee* OR labor* OR labour* OR staff* OR team* OR worker* OR workforce* OR profession* OR personnel*) OR TITLE-ABS-KEY (manpower OR "human resource*")	AND	TITLE-ABS-KEY (doctor* OR physician* OR practitioner* OR clinician* OR pharmacist* OR nurse* OR epidemiologist* OR "infection control practitioner*") OR TITLE-ABS-KEY (medical OR microbiology OR laboratory) AND (assistant* OR technician*)

c) Embase database

Search term				
**Antibiotics/antimicrobial resistance management**		**Human resource**		**Occupation**
('antimicrobial stewardship'/exp OR 'antibiotic stewardship'/exp OR ('antimicrobial' NEAR/3 'stewardship'):ab,ti OR ('antibiotic' NEAR/3 'stewardship'):ab,ti OR 'asp':ab,ti)	AND	('health manpower'/exp OR ('human' NEAR/2 ('resource' OR 'resources')):ab,ti OR ('full time equivalent' OR 'fte'):ab,ti OR ('staffing' OR 'workforce'):ab,ti OR 'professional role'/exp OR ('professional' NEAR/3 ('role' OR 'roles' OR 'activity' OR 'activities')):ab,ti)	AND	('infectious disease specialist'/exp OR ('infectious' NEAR/2 'disease' NEAR/3 'physician*'):ab,ti) OR ('clinical pharmacist'/exp OR ('infectious' NEAR/2 'disease' NEAR/3 'pharmacist*'):ab,ti OR ('id' NEAR/2 'pharmacist*'):ab,ti) OR ('infection control nurse'/exp OR ('infection' NEAR/2 'control' NEAR/3 'nurse*'):ab,ti) OR ('hospital epidemiologist'/exp OR ('hospital' NEAR/3 'epidemiologist*'):ab,ti) OR ('laboratory personnel'/exp OR 'laboratory technician'/exp OR 'medical technologist'/exp OR 'microbiologist'/exp OR 'microbiologist*':ab,ti OR ('laboratory' NEAR/3 ('personnel' OR 'technician*')):ab,ti)

d) CINAHL (EBSCOhost) database

Search term				
**Antibiotics/antimicrobial resistance management**		**Human resource**		**Occupation**
(MH "Antimicrobial Stewardship+") OR TI (("antimicrobial stewardship") OR ("antibiotic stewardship") OR ("antimicrobial" N3 "stewardship") OR ("antibiotic" N3 "stewardship")) OR AB (("antimicrobial stewardship") OR ("antibiotic stewardship") OR ("antimicrobial" N3 "stewardship") OR ("antibiotic" N3 "stewardship")) OR TI (asp) OR AB (asp)	AND	(MH "Health Personnel+") OR (MH "Staffing") OR (MH "Workforce") OR (MH "Professional Roles+") OR TI (("human resource") OR ("human resources") OR ("full time equivalent") OR (fte) OR (staffing) OR (workforce) OR (role) OR (roles) OR (activity) OR (activities)) OR AB (("human resource") OR ("human resources") OR ("full time equivalent") OR (fte) OR (staffing) OR (workforce) OR (role) OR (roles) OR (activity) OR (activities))	AND	(MH "Physicians, Infectious Disease") OR TI (("infectious disease" N3 physician*)) OR AB (("infectious disease" N3 physician*)) OR (MH "Pharmacists, Clinical") OR TI (("infectious disease" N3 pharmacist*) OR ("id" N2 pharmacist*)) OR AB (("infectious disease" N3 pharmacist*) OR ("id" N2 pharmacist*)) OR (MH "Nurses, Infection Control") OR (MH "Infection Control Practitioners+") OR TI (("infection control" N3 nurse*)) OR AB (("infection control" N3 nurse*)) OR (MH "Epidemiologists") OR TI (("hospital" N3 epidemiologist*)) OR AB (("hospital" N3 epidemiologist*)) OR (MH "Laboratory Personnel+") OR (MH "Technologists, Medical") OR (MH "Microbiologists") OR (MH "Laboratory Technicians") OR TI (

				(microbiologist*) OR ("medical technologist*") OR ("laboratory" N3 (personnel OR technician*))) OR AB ((microbiologist*) OR ("medical technologist*") OR ("laboratory" N3 (personnel OR technician*)))

e) CABI database

**Database**	**Search term**				
	**Antibiotics/antimicrobial resistance management**		**Human resource**		**Occupation**
CABI	anti-bacterial OR antibacterial OR anti-mycobacterial OR antimycobacterial OR anti-fungal OR antifungal OR anti-biotic OR antibiotic OR anti-microbial OR antimicrobial OR anti-infective OR anti-infective agents stewardship OR prevent OR management OR control OR Antimicrobial Stewardship	AND	health OR healthcare OR health care employee OR labor OR labour OR staff OR team OR worker OR workforce or personnel OR Manpower OR Human resource	AND	doctor OR physician OR practitioner OR clinician OR pharmacist OR nurse OR epidemiologist OR (medical OR microbiology OR laboratory OR infection control practitioners)

Table S2 Characteristics of included studies (n = 50); a) Role and activities on ASPs among five healthcare professionals (N=44), b) The studies of FTE on antibiotic stewardship program (N=7)

	Author/ Year	Healthcare professionals	Country	Income level	Setting	Method of study	Number of populations	Objective
**a) Role and activities on ASPs among five healthcare professionals (N=44)**								
	**1. Nurse**							
1.	Bos et al. (2024)	N	The Netherlands	High	Surgical or internal medicine ward in a hospital	A qualitative study	14	To explore the views and visions of Dutch bedside nurses on their role regarding appropriate antimicrobial use.
2.	van Gulik et al. (2021)	N	Thailand	upper-middle	1000-bed university hospital	A qualitative study	33	To explore how organisational multidisciplinary leaders and clinical nurses perceive nurses' roles in antimicrobial stewardship
3.	Dowson et al. (2020)	N	Australia	High	aged-care homes	A qualitative study	12	To investigate the potential opportunities for nurses to undertake antimicrobial stewardship activities near the end of life in aged-care homes

	Author/ Year	Healthcare professionals	Country	Income level	Setting	Method of study	Number of populations	Objective
4.	Olans et al. (2020)	N	N/A	-	N/A	Review	N/A	Review the structure and function of ASPs with particular attention to the role of nurses in the stewardship process.
5.	Abbas et al. (2019)	N	USA	High	Tertiary care academic center (860 beds)	A cross-sectional survey (n = 159 respondents)	159/3,485 (4.56%)	To determine the knowledge, attitudes, and practices of nursing staff members regarding ASPs and identify barriers to their participation in such programmes.
6.	Castro-Sánchez et al. (2019)	N	UK	High	Public sector organisation	Group discussion (n = 109 respondents)	N/A	To reflect upon the variety of antimicrobial stewardship nursing models already implemented in the UK.
7.	Wilcock et al (2019)	N	USA	High	Acute teaching hospital (750 beds)	An online survey (n = 80 respondents)	N/A	To determine the views of nurses and midwives in an acute hospital regarding a potential role in an antimicrobial stewardship programme.

	Author/ Year	Healthcare professionals	Country	Income level	Setting	Method of study	Number of populations	Objective
8.	Wiley and Villamizar (2019)	N	USA	High	Healthcare facilities	Review	N/A	To discuss the multiple aspects of antibiotic resistance, antibiotic stewardship, and nursing's role in improving patient care and promoting strong policy in the face of evolving antibiotic resistance.
9.	Rout and Brysiewicz (2017)	N	South Africa	Upper-middle	ICU in an acute private hospital (200 beds)	In-depth interview (n = 15 respondents)	N/A	To explore the perceptions of AMS team members regarding the role of the ICU nurse in the AMS team.
10.	Manning et al. (2016)	N	USA	High	Acute care hospital	Review (Practice forum)	N/A	To convey the need for nurses to combat antibiotic resistance.
11.	Olans et al. (2016)	N	N/A	-	N/A	Review (Invited article)	N/A	To evaluates nursing stewardship activities and analyzes the potential benefits of nurses' formal education.
12.	Glover. (2000)	N	N/A	-	Hospital	Review	N/A	To increase knowledge and understanding about how drug resistance organisms affect nursing.

	Author/ Year	Healthcare professionals	Country	Income level	Setting	Method of study	Number of populations	Objective
	**2. Pharmacist**							
1.	Umemura et al. (2022)	Rx	Japan	High	community hospitals	A cross-sectional study	2596 cases	To study the FTE on post-prescription review with feedback (PPRF) intervention.
2.	Abubakar and Tangiisuran (2020)	Rx	Nigeria	Lower-middle	Tertiary hospitals	A cross-sectional nationwide online survey (n = 37 hospitals)	37/45 (82.2%)	To evaluate the activities and barriers to hospital pharmacists' participation in antimicrobial stewardship programmes in Nigerian tertiary hospitals.
3.	Laible et al. (2019)	Rx	USA	High	Small private hospitals	Interview (n = 33 hospitals)	N/A	The stages of development of a health system–wide antimicrobial stewardship program using existing personnel and technology are described.
4.	Kullar et al, 2018 (30)	Rx	USA	High	Long-term care facilities (LTCFs)	Review (Special report)	N/A	To provide a roadmap on how to implement ASPs in LTCFs.

	Author/ Year	Healthcare professionals	Country	Income level	Setting	Method of study	Number of populations	Objective
5.	Garau and Bassetti (2018)	Rx	N/A	-	Hospital and community	Review	N/A	To review the role of pharmacists within AMS programmesand the opportunities for pharmacist-driven AMS strategies in hospital and community settings.
6.	Beach et al. (2017)	Rx	Canada	High	Tertiary care academic hospitals	A questionnaire by email (n = 68 respondents)	68/88 (77%)	To describe the demographic characteristics and roles of AMS teams in Canadian tertiary care academic hospitals.
7.	Echevarria et al. (2017)	Rx	USA	High	Veterans Health Administration facilities	Experiment (n = 12 hospitals)	N/A	The development and validation of a staffing calculator and its use in creating staffing guidance for antimicrobial stewardship programmes.
8.	Gilchrist et al. (2015)	Rx	UK	High	N/A	Review	N/A	To describes the evolution of the antimicrobial pharmacist role, its impact, the progress toward the actions in the UK.

	Author/ Year	Healthcare professionals	Country	Income level	Setting	Method of study	Number of populations	Objective
9.	Septimus et al. (2011)	Rx	USA	High	Community hospital	Review (Supplementary article)	N/A	To review the ASP strategies and resources currently available to community hospitals.
10.	Hand. (2007)	Rx	UK	High	Hospital	Review	N/A	Review examines the origins of the specialist antibiotic pharmacist and how the role has developed in recent years.
11.	Drummond. (2006)	Rx	UK	High	Acute care hospital	Meeting report	N/A	The focus of the meeting was the developing role of the antibiotic pharmacist.
	**3.Physician**							
1.	Avent et al. (2020)	P	USA	High	Primary care	Review	N/A	To discuss a top-down approach to ensure the sustainable implementation and uptake of AMS interventions in the community.

	Author/ Year	Healthcare professionals	Country	Income level	Setting	Method of study	Number of populations	Objective
2.	Binda et al. (2020)	P	France	High	Public and privateacute care hospitals (>50 beds)	A cross-sectional online survey (n = 97 hospitals)	97/215 (45%)	To evaluate the current state of antibiotic stewardship (ABS) in French public and private acute care hospitals.
3.	Xia et al. (2019)	P	China	Upper-middle	Respondents from all fields	Mobile phone Application survey (n = 1,194 respondents)	1,194/19,791 (6.03%)	To explore doctors' knowledge, willingness, concerns and the countermeasures to the antimicrobial stewardship regulations of China
4.	Bryant. (2015)	P	Australia and New Zealand	High	Tertiary paediatric hospital (40-300 beds)	Web-based survey (n = 14 hospitals)	N/A	To identify current antimicrobial stewardship resources and activities for children in hospitals, to identify gaps in services.
	**4. Clinical microbiologist**							
1.	Morency-Potvin et al. (2017)	T	N/A	-	Healthcare settings	Review	N/A	To provide a discussion of the components of antimicrobial stewardship in which microbiology laboratories can make significant contributions.

	Author/ Year	Healthcare professionals	Country	Income level	Setting	Method of study	Number of populations	Objective
2.	vanões et al. (2016)	T	N/A	-	N/A	Review	N/A	To address the role and importance of the microbiology laboratory in antibiotic stewardship
3.	Leuthner et al. (2013)	T	USA	High	Hospital	Review	N/A	This issue serves as the fundamental basis for the concept of antimicrobial stewardship.
4.	Peterson et al. (2001)	T	N/A	-	N/A	Review (Invited article)	N/A	To discuss the impact of microbiology laboratory practice on the management of infectious diseases–related health care issues.
	**5.Epidemiologist**							
1.	Abbas and Stevens (2018)	E	USA	High	Hospital	Review	N/A	To describe the role of the hospital epidemiologist in antibiotic stewardship.

	Author/ Year	Healthcare professionals	Country	Income level	Setting	Method of study	Number of populations	Objective
2.	Kaye et al. (2015)	E	N/A	-	N/A	Guideline	N/A	To outline an essential role of epidemiologist at times assumes and also details the pertinent skills and competencies.
3.	Moody et al. (2012)	E	USA	High	Acute, inpatient, long-term care, and outpatient settings.	Review (Position Paper)	N/A	To highlight the critical importance of health care epidemiologists in effective antimicrobial stewardship programs.
	**6.multi-sectoral healthcare team**							
1.	Cutrell and Sanders (2024)	P, Rx	USA	High	Acute care facility	A case study	1	To consider roles of antimicrobial stewards in interprofessional
2.	Logan et al. (2019)	P, Rx	USA	High	Acute care facility (<50 to 827 beds)	A gap analysis survey (n = 28 hospitals)	N/A	To describes the first year of forming a systemwide stewardship collaborative across a diverse health care system.

	Author/ Year	Healthcare professionals	Country	Income level	Setting	Method of study	Number of populations	Objective
3.	Cho et al. (2018)	P, Rx	USA	High	Community acute care hospitals	An electronic survey (n = 20 hospitals)	20/28 (71.4%)	To assess current ASPs and practices of acute care hospitals in East Texas.
4.	Doernber g et al. (2018)	P, Rx	USA	High	Acute care facility	A cross-sectional survey (n = 244 hospitals)	244/1989 (12%)	To describe results of a survey of US stewardship programmesand recommending potential staffing structures in the acute care setting.
5.	Gardiner et al. (2017)	P, Rx	New Zealand	High	Public hospitals	A quantitative survey (n = 20 hospitals)	20/20 (100%)	To determine what antimicrobial stewardship practices exist in New Zealand public hospitals.
6.	Le coz et al. (2016)	P, Rx , T	France	High	Healthcare facilities	An online cross-sectional nationwide survey (n = 65hospitals)	N/A	To evaluate the human resources needed to implement the multidisciplinary antimicrobial stewardship teams in French healthcare facilities.

	Author/ Year	Healthcare professionals	Country	Income level	Setting	Method of study	Number of populations	Objective
7.	Kim et al (2015)	P, Rx, T	USA	High	Teaching hospital	Review (Special report)	N/A	To review the need for antimicrobial stewardship and the key components of setting up a program
8.	Dellit et al. (2007)	P, Rx, T	USA	High	Acute care hospital	Guideline	N/A	The document presents guidelines for developing institutional programmesto enhance antimicrobial stewardship
9.	Macdougall and Polk (2005)	P, Rx, T, E	N/A	-	Hospitals	Review	N/A	To address the rationale, structure, analysis, and outcomes of antimicrobial stewardship programmeswith a special interest in their impact on antimicrobial resistance.
10.	Schellack et al. (2016)	Rx, N	South Africa	Upper-middle	Hospital	Review	N/A	The article proposed possible ways of engagement between the pharmacist, and nurse and doctor.
	**b) The studies of FTE on antibiotic stewardship program (N=7)**							

	Author/ Year	Healthcare professionals	Country	Income level	Setting	Method of study	Number of populations	Objective
1	Umemura et al. (2022)	FTEs of Rx	Japan	High	**Hospital type:** community hospitals	A cross-sectional study in one hospital	2596 cases	To study the FTE on post-prescription review with feedback (PPRF) intervention.
2.	Maeda et al. (2019a)	FTEs of P, Rx, N, T	Japan	High	**Hospital type:** Predominantly general hospitals (small, medium, large hospitals) with not explicitly stratified hospital type	Nationwide survey (n = 1,358 hospitals)	1,358/3,532 (38.4%)	To report the potential staffing structures for ASPs proposed based on a nationwide survey.
3.	Maeda et al. (2019b)	FTEs of P, Rx, N,T	Japan	High	**Hospital type:** Predominantly general hospitals (small, medium, large hospitals) with not explicitly stratified hospital type	A paper-based survey (n = 1,358 hospitals)	1,358/3,532 (38.4%)	To clarify what percentage of hospitals nationwide is implementing ASPs and staffing resources.
4.	Nhan et al (2019)	FTEs of P, Rx, N, E	USA	High	**Hospital type:** academic or tertiary centres	An online survey (n = 101 hospitals)	101/200 (51%)	To examine antibiotic stewardship programme structure among high-performing hospitals

	Author/ Year	Healthcare professionals	Country	Income level	Setting	Method of study	Number of populations	Objective
5.	Kallen et al. (2018)	FTEs of P, Rx, N, T, E	Netherland	High	**Hospital type:**<br>-University hospitals: 8%<br>-Non-university teaching hospitals: 58%<br>-General non-teaching hospitals: 34%	Survey (n = 64 hospitals)	64/80 (80%)	To evaluate the current state of antimicrobial stewardship in hospitals.
6.	Doernberg et al. (2018)	FTEs of P, Rx	USA	High	**Hospital type:**<br>- Academic centres: 21%<br>- Academically affiliated hospitals: 46%<br>-Others: remainder	A cross-sectional survey (n = 244 hospitals)	244/1989 (12%)	To describe results of a survey of US stewardship programmes and recommending potential staffing structures in the acute care setting.
7.	Gardiner et al. (2017)	FTEs of P, Rx	New Zealand	High	**Hospital type:**<br>Public hospitals with not explicitly stratified hospital type	A quantitative survey (n = 20 hospitals)	20/20 (100%)	To determine what antimicrobial stewardship practices exist in New Zealand public hospitals.

Noted: The meta-analysis was limited to studies that reported the number of participants of FTE antimicrobial stewardship professionals and standard error among five healthcare professionals.

Table S3 Core functions and activities in the ASPs of ID physicians (n = 13)

	Core functions and activities	Cutrell et al (2024)	Avent et al (2020)	Binda et al (2020)	Logan et al (2019)	Xia et al (2019)	Cho et al (2018)	Doernberg et al (2018)	Gardiner et al (2017)	Le coz et al (2016)	Bryant et al (2015)	Kim et al (2015)	Dellit et al (2007)	Macdougall et al (2005)	total
	**1. Prescribing oversight and policy leadership**														
1	Developing clinical guidelines			✓	✓	✓	✓	✓	✓	✓	✓	✓	✓	✓	11
2	Implementing formulary restrictions		✓	✓		✓	✓	✓	✓		✓	✓	✓		9
3	Documenting written policies and institutional commitments		✓	✓	✓	✓	✓		✓	✓		✓			8
4	Utilising antimicrobial order forms			✓			✓				✓	✓	✓		5
5	Developing and maintaining antibiograms						✓			✓					2
6	Participating in ASP network activities				✓	✓				✓					3
	**2. Antimicrobial optimisation (clinical management)**				□	□				□					
7	Implementing antimicrobial cycling strategies			✓			✓				✓		✓	✓	5
8	De-escalating antimicrobial therapy based on clinical and microbiological data			✓	✓		✓				✓		✓		5
9	Switching intravenous antibiotics to oral formulations when appropriate			✓	✓		✓				✓		✓		5
10	Using combination therapy when clinically indicated			✓	✓		✓				✓		✓		5
11	Optimising antimicrobial dosing			✓	✓		✓				✓		✓		5
12	Discontinuing unnecessary or inappropriate antibiotics			✓	✓		✓	✓			✓		✓		6

	Core functions and activities	Cutrell et al (2024)	Avent et al (2020)	Binda et al (2020)	Logan et al (2019)	Xia et al (2019)	Cho et al (2018)	Doernberg et al (2018)	Gardiner et al (2017)	Le coz et al (2016)	Bryant et al (2015)	Kim et al (2015)	Dellit et al (2007)	Macdougall et al (2005)	total
13	Conducting personalised medical chart reviews and ward rounds	✓		✓					✓	✓		✓			5
14	Implementing daily care bundles to optimise antibiotic use			✓											1
	**3. Monitoring, audit, and feedback**			□											
15	Conducting ward-level/hospital-wide audits										✓				1
16	Conducting prospective audits with feedback		✓	✓	✓		✓	✓	✓	✓	✓	✓	✓		10
17	Monitoring and reporting antimicrobial consumption data		✓	✓	✓		✓		✓	✓	✓				7
18	Tracking progress toward stewardship implementation goals				✓		✓		✓	✓		✓			5
19	Reporting antimicrobial use and resistance information to clinical staff				✓					✓	✓				3
20	Overseeing antimicrobial adherence and providing bedside consultation			✓						✓				✓	3
	**4. Decision-support and diagnostics integration**										□				
21	Using computerised order entry systems to support antimicrobial decision-making											✓			1
22	Managing computer-assisted systems for patient-specific antimicrobial recommendations		✓					✓		✓		✓		✓	5
23	Providing point-of-care antimicrobial susceptibility reports		✓	✓		✓		✓							4

	Core functions and activities	Cutrell et al (2024)	Avent et al (2020)	Binda et al (2020)	Logan et al (2019)	Xia et al (2019)	Cho et al (2018)	Doernberg et al (2018)	Gardiner et al (2017)	Le coz et al (2016)	Bryant et al (2015)	Kim et al (2015)	Dellit et al (2007)	Macdougall et al (2005)	total
24	Supporting clinical diagnostic tests for pathogen detection				✓	✓					✓	✓			4
	**5. Education and capacity building**				□	□					□	□			
25	Providing education for medical staff	✓	✓		✓				✓	✓	✓	✓	✓	✓	9
26	Providing education and consultation services for patients		✓						✓				✓		3
27	Conducting ongoing training on antimicrobial-related programs			✓	✓	✓				✓	✓			✓	6

Remarks:The symbol ✓indicated an article describing the antimicrobial stewardship roles and activities in each topic.

Table S4 Core functions and activities in the ASPs of ID clinical pharmacists (n = 21)

	Core functions and activities	Cutrell et al (2024)	Umemura et al (2022)	Abubakar et al (2020)	Laible et al (2019)	Logan et al (2019)	Cho et al. (2018)	Doernberg et al (2018)	Garau and Baseetti (2018)	Beach et al (2017)	Echevarria et al (2017)	Gardiner et al (2017)	Kullar et al (2018)	Le coz et al (2016)	Schellack et al (2016)	Gilchrist et al (2015)	Kim et al (2015)	Septimus et al (2011)	Dellit et al (2007)	Hand et al (2007)	Drummond et al (2006)	Macdougall et al (2005)	total
	**1. Prescribing oversight and policy leadership**																						
1	Developing clinical guidelines			✓		✓	✓	✓	✓	✓		✓	✓	✓		✓	✓	✓	✓	✓	✓	✓	16
2	Developing and maintaining antibiograms						✓			✓	✓		✓	✓		✓							6
3	Implementing formulary restrictions	✓		✓	✓		✓	✓	✓	✓	✓	✓				✓	✓		✓	✓			13
4	Documenting written policies and institutional commitments				✓	✓	✓		✓	✓		✓	✓	✓		✓	✓			✓			11
5	Utilising antimicrobial order forms						✓			✓									✓	✓			4
6	Participating in ASP network activities					✓			✓				✓	✓									4
	**2. Antimicrobial optimisation (clinical intervention)**					□			□				□	□									
7	Implementing antimicrobial cycling strategies						✓			✓							✓		✓	✓			5
8	De-escalating antimicrobial therapy based on clinical and microbiological data		✓	✓	✓	✓	✓			✓	✓							✓	✓	✓			10

	Core functions and activities	Cutrell et al (2024)	Umemura et al (2022)	Abubakar et al (2020)	Laible et al (2019)	Logan et al (2019)	Cho et al. (2018)	Doernberg et al (2018)	Garau and Baseetti (2018)	Beach et al (2017)	Echevarria et al (2017)	Gardiner et al (2017)	Kullar et al (2018)	Le coz et al (2016)	Schellack et al (2016)	Gilchrist et al (2015)	Kim et al (2015)	Septimus et al (2011)	Dellit et al (2007)	Hand et al (2007)	Drummond et al (2006)	Macdougall et al (2005)	total
9	Switching intravenous antibiotics to oral formulations when appropriate		✓	✓	✓	✓	✓		✓	✓	✓								✓	✓			10
10	Using combination therapy when clinically indicated				✓	✓	✓			✓	✓								✓	✓			7
11	Optimising antimicrobial dosing		✓	✓	✓	✓	✓			✓	✓							✓	✓	✓			10
12	Discontinuing unnecessary or inappropriate antibiotics	✓		✓	✓	✓	✓	✓	✓	✓	✓							✓	✓	✓			12
13	Implementing daily care bundles to optimise antibiotic use				✓						✓							✓					3
14	Assessing the appropriateness of antimicrobial prescriptions		✓	✓					✓	✓			✓		✓	✓		✓				✓	9
15	Conducting personalised medical chart reviews and ward rounds			✓					✓	✓		✓		✓		✓	✓						7
16	Applying pharmacokinetic principles and performing therapeutic drug monitoring				✓				✓		✓				✓					✓			5
17	Face-to-face/telemedicine consultation				✓				✓	✓	✓		✓	✓	✓	✓				✓		✓	10
	**3. Monitoring, audit, and feedback**				□						□							□					

	Core functions and activities	Cutrell et al (2024)	Umemura et al (2022)	Abubakar et al (2020)	Laible et al (2019)	Logan et al (2019)	Cho et al. (2018)	Doernberg et al (2018)	Garau and Baseetti (2018)	Beach et al (2017)	Echevarria et al (2017)	Gardiner et al (2017)	Kullar et al (2018)	Le coz et al (2016)	Schellack et al (2016)	Gilchrist et al (2015)	Kim et al (2015)	Septimus et al (2011)	Dellit et al (2007)	Hand et al (2007)	Drummond et al (2006)	Macdougall et al (2005)	total
18	Conducting ward-level/hospital-wide audits			✓																			1
19	Conducting prospective audits with feedback		✓	✓	✓	✓	✓	✓	✓	✓	✓	✓	✓	✓		✓	✓	✓	✓	✓			16
20	Monitoring and reporting antimicrobial consumption data		✓	✓	✓	✓	✓		✓	✓	✓	✓	✓	✓	✓	✓					✓		13
21	Tracking progress toward stewardship implementation goals			✓	✓	✓	✓		✓	✓	✓	✓	✓	✓	✓	✓	✓						13
22	Reporting antimicrobial use and resistance information to clinical staff			✓	✓	✓							✓	✓				✓		✓	✓		8
	**4. Decision-support and diagnostics integration**			□																			
23	Using computerised order entry systems to support antimicrobial decision-making								✓	✓	✓		✓			✓	✓			✓			7
24	Managing computer-assisted systems for patient-specific antimicrobial recommendations							✓			✓		✓	✓		✓	✓					✓	7
25	Providing point-of-care antimicrobial susceptibility reports				✓			✓			✓												3

	Core functions and activities	Cutrell et al (2024)	Umemura et al (2022)	Abubakar et al (2020)	Laible et al (2019)	Logan et al (2019)	Cho et al. (2018)	Doernberg et al (2018)	Garau and Baseetti (2018)	Beach et al (2017)	Echevarria et al (2017)	Gardiner et al (2017)	Kullar et al (2018)	Le coz et al (2016)	Schellack et al (2016)	Gilchrist et al (2015)	Kim et al (2015)	Septimus et al (2011)	Dellit et al (2007)	Hand et al (2007)	Drummond et al (2006)	Macdougall et al (2005)	total
26	Supporting clinical diagnostic tests for pathogen detection				✓	✓											✓						3
27	Coordinating antimicrobial-related care with external hospitals										✓												1
	**5. Education and capacity building**										□												
28	Providing education for medical staff			✓		✓				✓	✓	✓	✓	✓	✓	✓	✓		✓	✓	✓	✓	14
29	Providing education and consultation services for patients			✓		✓				✓	✓	✓	✓						✓				7
30	Conducting ongoing training on antimicrobial-related programs				✓	✓			✓				✓	✓								✓	6
31	Delivering educational printed or online materials				✓								✓										2
32	Participating in research									✓					✓	✓				✓			4

Table S5 Core functions and activities in the ASPs for ICNs (n = 13)

	Core functions and activities	Bos et al (2024)	van Gulik et al (2021)	Dowson et al (2020)	Olans et al (2020)	Abbas et al (2019)	Castro-Sánchez et al (2019)	Wilcock et al (2019)	Wiley et al (2019)	Rout et al (2017)	Manning et al (2016)	Olans et al (2016)	Schellack et al (2016)	Glover et al (2000)	total
	**1. Prescribing oversight and policy adherence**														
1	Ensuring accurate recording of antibiotic indications and treatment durations	✓	✓			✓	✓			✓				✓	6
2	Ensuring adherence to antimicrobial prescribing guidelines						✓	✓		✓			✓	✓	5
3	Advocating for AMS program and intervention						✓				✓				2
	**2. Antimicrobial optimisation (clinical and bedside practice)**														
4	Assessing and verifying patient allergy histories		✓		✓	✓	✓					✓	✓		6
5	Adjusting antimicrobial dosing and performing bedside de-escalation	✓			✓	✓	✓			✓	✓	✓	✓	✓	9
6	Switching intravenous antibiotics to oral formulations when appropriate	✓	✓		✓	✓	✓	✓				✓	✓		8
7	Obtaining early and appropriate microbiological cultures				✓	✓		✓				✓		✓	5

	Core functions and activities	Bos et al (2024)	van Gulik et al (2021)	Dowson et al (2020)	Olans et al (2020)	Abbas et al (2019)	Castro-Sánchez et al (2019)	Wilcock et al (2019)	Wiley et al (2019)	Rout et al (2017)	Manning et al (2016)	Olans et al (2016)	Schellack et al (2016)	Glover et al (2000)	total
8	Performing medication reconciliation to ensure accurate antimicrobial therapy				✓	✓						✓			3
9	Initiating antibiotics in a timely and clinically appropriate manner	✓	✓		✓	✓	✓			✓		✓	✓	✓	9
10	Implementing daily care bundles to optimise antibiotic use	✓						✓		✓			✓		4
11	Adjusting antimicrobial therapy based on final culture results	✓	✓		✓	✓	✓	✓		✓		✓		✓	9
	**3. Monitoring, audit, and feedback**	□	□		□	□	□	□		□		□		□	
12	Monitoring and reporting patient progress relevant to antimicrobial therapy	✓	✓		✓	✓	✓			✓		✓	✓	✓	9
13	Reviewing preliminary culture results to guide timely antimicrobial adjustment	✓	✓		✓	✓		✓		✓		✓		✓	8
14	Reviewing changes in patient clinical condition affecting antimicrobial decisions	✓	✓	✓	✓					✓		✓	✓	✓	8
15	Monitoring adverse events related to antimicrobial therapy	✓	✓		✓					✓		✓	✓	✓	7
16	Managing outpatient antimicrobial therapy				✓							✓			2
17	Coordinating long-term antimicrobial management and monitoring readmission risk		✓		✓							✓			3

	Core functions and activities	Bos et al (2024)	van Gulik et al (2021)	Dowson et al (2020)	Olans et al (2020)	Abbas et al (2019)	Castro-Sánchez et al (2019)	Wilcock et al (2019)	Wiley et al (2019)	Rout et al (2017)	Manning et al (2016)	Olans et al (2016)	Schellack et al (2016)	Glover et al (2000)	total
	**4. Communication, coordination, and multidisciplinary collaboration**		□		□							□			
18	Performing patient triage and ensuring appropriate isolation practices				✓	✓						✓		✓	4
19	Conducting personalised medical chart reviews and ward rounds									✓			✓		2
20	Participating in regular multidisciplinary clinical quality meetings									✓	✓				2
21	Identifying potential antimicrobial resistance during patient care									✓		✓			2
	**5. Education and capacity building**									□		□			
22	Providing education for medical staff								✓		✓				2
23	Providing education and consultation services for patients	✓	✓	✓	✓	✓	✓	✓	✓		✓	✓		✓	11
24	Providing education for the public						✓	✓			✓				3
25	Raising awareness of antimicrobial stewardship activities							✓	✓		✓				3

Table S6 Core functions and activities in the ASPs of clinical microbiologists (n = 8)

	Core functions and activities	Morency-Potvin et al (2017)	Le coz et al (2016)	Simões et al (2016)	Kim et al (2015)	Leuthner et al (2013)	Peterson et al (2001)	Dellit et al (2007)	Macdougall et al (2005)	total
	**1. Prescribing oversight and policy leadership**									
1	Developing clinical guidelines	✓		✓			✓		✓	4
2	Establishing guidelines for specimen collection and processing	✓		✓						2
3	Providing guidance on diagnostic testing and antimicrobial therapy	✓	✓	✓	✓		✓	✓	✓	7
	**2. Diagnostic stewardship and laboratory optimisation**									
4	Optimising routing and tracking of specimens to the laboratory	✓					✓			2
5	Supporting the use of computerised order entry systems for diagnostic testing			✓						1
6	Performing rapid diagnostic testing for pathogens and clinical specimens	✓			✓		✓			3
7	Ensuring availability of appropriate diagnostic testing	✓			✓		✓			3
8	Promoting the appropriate use of point-of-care microbiological tests	✓			✓					2
	**3. Surveillance, monitoring, and reporting**	□			□					
9	Reporting antimicrobial use and resistance information to clinical staff	✓	✓	✓	✓		✓	✓	✓	7

	Core functions and activities	Morency-Potvin et al (2017)	Le coz et al (2016)	Simões et al (2016)	Kim et al (2015)	Leuthner et al (2013)	Peterson et al (2001)	Dellit et al (2007)	Macdougall et al (2005)	total
10	Implementing cascade reporting of susceptibility results	✓	✓	✓	✓					4
11	Developing and disseminating annual reports	✓		✓	✓	✓	✓	✓	✓	7
12	Conducting surveillance for emerging pathogens and antimicrobial resistance patterns	✓		✓	✓	✓	✓	✓	✓	7
13	Implementing internal and external quality control procedures to ensure laboratory accuracy			✓			✓			2
14	Participating in national and regional AMR surveillance systems	✓	✓	✓	✓		✓			5
15	Collaborating in audit and feedback on antimicrobial therapy for specific pathogens or syndromes	✓	✓	✓	✓		✓			5
	**4. Communication and multidisciplinary collaboration**	□	□	□	□		□			
16	Maintaining direct and indirect communication channels with prescribers	✓	✓	✓	✓		✓			5
17	Participating in personalised medical chart reviews and multidisciplinary ward rounds		✓							1
18	Ensuring availability of appropriate specimen disposal systems	✓								1
	**5. Education and capacity building**	□								
19	Providing education for medical staff	✓	✓	✓			✓			4
21	Providing continuous on-the-job training on endorsed laboratory methods			✓	✓					2

	Core functions and activities	Morency-Potvin et al (2017)	Le coz et al (2016)	Simões et al (2016)	Kim et al (2015)	Leuthner et al (2013)	Peterson et al (2001)	Dellit et al (2007)	Macdougall et al (2005)	total
22	Providing education for the public		✓	✓						2

Table S7 Core functions and activities in the ASPs of hospital epidemiologists (n = 4)

	Core functions and activities	Abbas et al (2018)	Kaye et al (2015)	Moody et al (2012)	Macdougall et al (2005)	total
	**1. Prescribing oversight and policy leadership**					
1	Developing clinical guidelines		✓	✓	✓	3
2	Developing and maintaining antibiograms	✓	✓	✓		3
3	Implementing formulary restrictions	✓				1
4	Providing guidance on diagnostic testing and antimicrobial therapy	✓	✓	✓		3
5	Developing infection prevention and control practices	✓	✓	✓	✓	4
	**2. Surveillance, monitoring, and reporting**	□	□	□		
6	Using computerised order entry systems to support surveillance and monitoring processes	✓		✓		2
7	Interpreting and implementing surveillance activities related to healthcare-associated infections		✓	✓	✓	3
8	Applying appropriate patient isolation precautions		✓	✓		2
9	Collecting and reporting normalised antimicrobial use data	✓	✓	✓		3
10	Sharing up-to-date institution-specific antimicrobial resistance data	✓	✓	✓		3
11	Developing and presenting reports on interventions, cost savings, and patient safety outcomes		✓	✓	✓	3
12	Using benchmarking data to assess institutional performance and identify areas for improvement		✓			1
	**3. Communication and multidisciplinary collaboration**		□			
13	Collaborating with other healthcare professionals on infection prevention initiatives	✓	✓	✓		3
14	Participating in personalised chart reviews and multidisciplinary ward rounds	✓				1
15	Maintaining direct and indirect communication channels with prescribers	✓				1
16	Conducting regular clinical quality meetings with multidisciplinary teams	✓				1
	**4. Education and capacity building**	□				
17	Providing education for medical staff	✓	✓	✓		3

	Core functions and activities	Abbas et al (2018)	Kaye et al (2015)	Moody et al (2012)	Macdougall et al (2005)	total
18	Providing education and consultation services for patients	✓				1
19	Providing education for the public	✓	✓			2
20	Participating in research		✓			1

Table S8 The number of FTEs of ASPs among five healthcare professionals (N=7)

Study id	Author	Year	Health professional	No. of beds	N (No. of population)	Mean (FTE)	SD	Qualit y score	
1	Maeda et al.	2019a	Physician	<100	294	0.1	0.1	57.1	Medium
2	Maeda et al.	2019a	Physician	100-300	552	0.1	0.05	57.1	Medium
3	Maeda et al.	2019a	Physician	301-500	320	0.1	0.1	57.1	Medium
4	Maeda et al.	2019a	Physician	501-1000	192	0.25	0.3	57.1	Medium
5	Nhan et al.	2019	Physician	Undefined	101	0.5	1.5	57.1	Medium
6	Doernberg et al.	2018	Physician	<100	15	0.27	0.435	57.1	Medium
7	Doernberg et al.	2018	Physician	100-300	91	0.24	0.6	57.1	Medium
8	Doernberg et al.	2018	Physician	301-500	82	0.26	0.5	57.1	Medium
9	Kallen et al.	2018	Physician	301-500	64	0.1	0.2	57.1	Medium
10	Doernberg et al.	2018	Physician	501-1000	45	0.37	0.5	28.6	Low
11	Doernberg et al.	2018	Physician	>1000	11	0.46	0.6	57.1	Medium
12	Gardiner et al.	2017	Physician	<100	20	0.1	0.125	57.1	Medium
13	Maeda et al.	2019a	Pharmacist	<100	294	0.1	0.1	57.1	Medium
14	Maeda et al.	2019a	Pharmacist	100-300	552	0.1	0.075	57.1	Medium
15	Maeda et al.	2019a	Pharmacist	301-500	320	0.2	0.075	28.6	Low
16	Maeda et al.	2019a	Pharmacist	501-1000	192	0.5	0.125	57.1	Medium
17	Nhan et al.	2019	Pharmacist	Undefined	101	0.755	1.495	57.1	Medium
18	Doernberg et al	2018	Pharmacist	<100	15	0.61	1	57.1	Medium
19	Doernberg et al	2018	Pharmacist	100-300	91	0.63	1	57.1	Medium
20	Doernberg et al	2018	Pharmacist	301-500	82	0.89	1.5	57.1	Medium
21	Kallen et al	2018	Pharmacist	301-500	64	0.1	0.6	28.6	Low
22	Doernberg et al	2018	Pharmacist	501-1000	45	1.2	1	71.4	High
23	Doernberg et al	2018	Pharmacist	>1000	11	1.5	1.3	71.4	High
24	Gardiner et al.	2017	Pharmacist	<100	20	0.3	0.1	71.4	High
25	Maeda et al.	2019a	Nurse	<100	294	0.1	0.05	57.1	Medium
26	Maeda et al.	2019a	Nurse	100-300	552	0.1	0.1	71.4	High
27	Maeda et al.	2019a	Nurse	301-500	320	0.25	0.1	71.4	High
28	Maeda et al.	2019a	Nurse	501-1000	192	0.2	0.2	71.4	High
29	Nhan et al.	2019	Nurse	Undefined	101	0	1	71.4	High
30	Kallen et al.	2018	Nurse	301-500	64	0.4	0.85	71.4	High
31	Nhan et al.	2019	Epidemiologist	Undefined	101	0	1.5	57.1	Medium
32	Kallen et al.	2018	Epidemiologist	301-500	64	0.1	0.05	71.4	High
33	Maeda et al.	2019a	Clinical microbiologist	<100	294	0	0.1	71.4	High

34	Maeda et al.	2019a	Clinical microbiologist	100-300	552	0.1	0.075	57.1	Medium
35	Maeda et al.	2019a	Clinical microbiologist	301-500	320	0.1	0.075	57.1	Medium
36	Kallen et al.	2019	Clinical microbiologist	301-500	64	0.2	0.95	14.3	Low
37	Maeda et al.	2019a	Clinical microbiologist	501-1000	192	0.2	0.2	14.3	Low

Table S9 Quality assessment of included studies

a) **Results of critical appraisal of cross-sectional studies (N=17)**

	Author (year)	1. Were the criteria for inclusion in the sample clearly defined?	2. Were the study subjects and the setting described in detail?	3. Was the exposure measured in a valid and reliable way?	4. Were objective, standard criteria used for measurement of the condition?	5. Were confounding factors identified?	6. Were strategies to deal with confounding factors stated?	7. Were the outcomes measured in a valid and reliable way?	8. Was appropriate statistical analysis used?	Quality score	
1	Abubakar et al. (2020)	Y	Y	N	NA	N	N	Y	Y	57.1	Medium
2	Umemura et al. (2022)	Y	Y	Y	Y	NA	NA	Y	Y	100	High
3	Abbas et al. (2019)	N	Y	N	NA	N	N	Y	N	28.6	Low
4	Binda et al. (2019)	Y	Y	N	NA	U	N	Y	N	42.9	Low
5	Logan et al. (2019)	N	Y	N	NA	N	N	Y	N	28.6	Low
6	Maeda et al. (2019a)	Y	Y	N	NA	N	N	Y	Y	57.1	Medium
7	Nhan et al. (2019)	N	Y	N	NA	N	N	Y	N	28.6	Low
8	Xia et al. (2019)	N	Y	N	NA	N	N	Y	Y	42.9	Low
9	Cho et al. (2018)	Y	Y	N	NA	Y	N	Y	Y	71.4	High
10	Wilcock et al. (2019)	N	N	N	NA	N	N	Y	N	14.3	Low
11	Abbas et al. (2018)	N	Y	N	NA	N	N	Y	N	28.6	Low
12	Doernberg et al. (2018)	Y	Y	N	NA	Y	Y	U	Y	71.4	High
13	Morris et al. (2018)	N	Y	N	NA	N	N	Y	Y	42.9	Low

	Author (year)	1. Were the criteria for inclusion in the sample clearly defined?	2. Were the study subjects and the setting described in detail?	3. Was the exposure measured in a valid and reliable way?	4. Were objective, standard criteria used for measurement of the condition?	5. Were confounding factors identified?	6. Were strategies to deal with confounding factors stated?	7. Were the outcomes measured in a valid and reliable way?	8. Was appropriate statistical analysis used?	Quality score	
14	Beach et al. (2017)	Y	Y	N	NA	N	N	Y	Y	57.1	Medium
15	Gardiner et al. (2017)	N	N	N	NA	N	N	Y	N	14.3	Low
16	Le coz et al. (2016)	N	Y	N	NA	N	N	Y	Y	42.9	Low
17	Bryant et al. (2015)	N	Y	N	NA	N	N	Y	N	28.6	Low
Note: Y: Yes, N: No, NA: Not applicable, U: unclear											

Note: Calculating the Quality Score (Percentage Method)
Calculate the percentage of "Yes" answers. For scoring purposes, "No" and "Unclear" are typically treated as the same (i.e., the criterion was not met). "Not Applicable" (NA) responses are excluded from the calculation.
The formula is: Quality Score = (Number of "Yes" responses) / (Total Questions - Number of "NA" responses) * 100%

Setting Quality Thresholds
High Quality: > 70%, Medium Quality: 50% – 69%, Low Quality: < 50%

b) Results of critical appraisal of qualitative study (N=1)

Item No	CASP Checklist questions	Rout and Brysiewicz, 2017
1	Was there a clear statement of the aims of the research?	Y
2	Is a qualitative methodology appropriate?	Y
3	Was the research design appropriate to address the aims of the research?	Y
4	Was the recruitment strategy appropriate to the aims of the research?	Y
5	Was the data collected in a way that addressed the research issue?	Y
6	Has the relationship between researcher and participants been adequately considered?	Y
7	Have ethical issues been taken into consideration?	Y
8	Was the data analysis sufficiently rigorous?	Y
9	Is there a clear statement of findings?	Y
10	How valuable is the research?	Y
Note: Y: Yes		

c) Results of critical appraisal of guideline (N=1)

AGREE II checklist questions	Dellit et al, 2007
**Domain 1: Scope and purpose**	
1. The overall objective(s) of the guideline is (are) specifically described.	7
2. The health question(s) covered by the guideline is (are) specifically described.	1
3. The population (patients, public, etc.) to whom the guideline is meant to apply is specifically described	7
**Domain 2: Stakeholder involvement**	
4. The guideline development group includes individuals from all relevant professional groups	6
5. The views and preferences of the target population (patients, public, etc.) have been sought.	6
6. The target users of the guideline are clearly defined	6
**Domain 3: Rigour of development**	
7. Systematic methods were used to search for evidence.	3
8. The criteria for selecting the evidence are clearly described	1
9. The strengths and limitations of the body of evidence are clearly described.	3
10. The methods for formulating the recommendations are clearly described.	1
11. The health benefits, side effects, and risks have been considered in formulating the recommendations	5
12. There is an explicit link between the recommendations and the supporting evidence.	5
13. The guideline has been externally reviewed by experts prior to its publication	1
14. A procedure for updating the guideline is provided	1

AGREE II checklist questions	Dellit et al, 2007
**Domain 4: Clarity for presentation**	
15. The recommendations are specific and unambiguous	4
16. The different options for management of the condition or health issue are clearly presented.	6
17. Key recommendations are easily identifiable.	4
**Domain 5: Applicability**	
18. The guideline describes facilitators and barriers to its application	4
19. The guideline provides advice and/or tools on how the recommendations can be put into practice	5
20. The potential resource implications of applying the recommendations have been considered.	5
21. The guideline presents monitoring and/or auditing criteria.	1
**Domain 6: Editorial independence**	
22. The views of the funding body have not influenced the content of the guideline	1
23. Competing interests of guideline development group members have been recorded and addressed	1
**Summarized score in each domain (Mean%)**	**Dellit et al, 2007**
Scope and purpose	67
Stakeholder involvement	83
Rigour of development	25
Clarity for presentation	61

AGREE II checklist questions	Dellit et al, 2007
Applicability	61
Editorial independence	0

Note: An "overall quality ranking" was not derived, and their scores were used for descriptive purposes only. AGREE-II assessment tools for guideline. We used the resulting 'Domain (Mean%)' scores.

d) Results of reporting quality of narrative studies (N=21)

	Author, year	1. Justification of articles importance for readership	2. Statement of concrete aims for formulation of questions	3. Description of the literature search	4 Referencing	5 Scientific reasoning	6. Appropriate presentation of data	Mean sum score for each study
1	Avent et al. (2020)	2	2	2	2	1	2	1.83
2	Kim et al. (2015)	2	2	0	1	1	2	1.33
3	Macdougall and Polk (2005)	2	2	0	1	2	1	1.33
4	Garau and Basseti (2018)	2	2	0	2	1	1	1.33
5	Kullar et al. (2017)	2	2	0	2	1	1	1.33
6	Schellack et al. (2016)	0	0	0	2	1	1	0.67
7	Gilchrist et al. (2015)	2	2	0	2	1	2	1.50
8	Septimus and Owens (2011)	1	2	0	1	1	2	1.17
9	Hand (2007)	2	2	0	2	2	2	1.67
10	Olans et al. (2020)	2	2	0	2	2	2	1.67

	Author, year	1. Justification of articles importance for readership	2. Statement of concrete aims for formulation of questions	3. Description of the literature search	4 Referencing	5 Scientific reasoning	6. Appropriate presentation of data	Mean sum score for each study
11	Wiley and Villamizar (2019)	2	2	0	2	1	2	1.50
12	Manning et al. (2016)	2	2	0	1	1	2	1.33
13	Olans et al. (2016)	2	0	0	2	1	2	1.17
14	Glover et al. (2000)	1	2	0	1	1	1	1.00
15	Morency-Potvin et al. (2017)	2	2	0	2	1	1	1.33
16	Simões et al. (2016)	2	2	0	2	1	2	1.50
17	Leuthner and Doern (2013)	2	2	0	1	1	1	1.17
18	Peterson et al. (2001)	1	1	0	1	1	1	0.83
19	Moody et al. (2012)	1	1	0	1	1	NA	0.80
20	Greene et al. (2019)	2	2	0	2	2	2	1.67
21	Morris et al. (2018)	2	2	2	2	2	2	2.00
	**Mean sum score for each item**	1.71	1.71	0.19	1.62	1.24	1.60	

Note: An "overall quality ranking" was not derived, and their scores were used for descriptive purposes only. SANRA assessment tools for narrative reviews): We used the resulting 'Mean sum score' (out of 2).

Table S10 Summary pooled for main and sensitivity analysis

No.	Infectious disease management team	Type analysis	No. of sub-studies	No. of participants	Mean of FTE	95% CI
**1**	**Infectious disease physicians**	**Main analysis**	**12**	**1787**	**0.14**	**0.12-0.16**
2	Infectious disease physicians	Sensitivity analysis	11	1767	0.14	0.12-0.17
**3**	**Infectious disease clinical pharmacists**	**Main analysis**	**12**	**1873**	**0.38**	**0.3-0.47**
4	Infectious disease clinical pharmacists	Sensitivity analysis	10	1752	0.37	0.28-0.47
**5**	**Infection control nurses**	**Main analysis**	**6**	**1523**	**0.18**	**0.12-0.25**
6	Infection control nurses	Sensitivity analysis	5	1422	0.18	0.11-0.25
**7**	**Clinical microbiologists**	**Main analysis**	**5**	**1422**	**0.11**	**0.05-0.17**
8	Clinical microbiologists	Sensitivity analysis	NA	NA	NA	NA
**9**	**Hospital Epidemiologists**	**Main analysis**	**2**	**165**	**0.1**	**0.08-0.12**
10	Hospital Epidemiologists	Sensitivity analysis	NA	NA	NA	NA

CI: Confidence interval; FTE: full-time equivalent, NA: Not applicable

Table S11 Assessment of publication bias

No.	Infectious disease management team	No. of studies	P value of Egger test	Funnel plot	Contour test		Conclusion
					Significant area	Non-significant area	
1	Infectious disease physicians	12	< 0.001	Asymmetry	Yes	No	Publication bias
2	Infectious disease clinical pharmacists	12	< 0.001	Asymmetry	Yes	No	Publication bias
3	Infectious control nurses	6	0.1158	Asymmetry	Yes	No	Inconclusive
4	Clinical microbiologists	5	0.1187	Asymmetry	Yes	No	Inconclusive
5	Hospital epidemiologists	2	0.3159	No	NA	NA	Inconclusive

NA: Not applicable

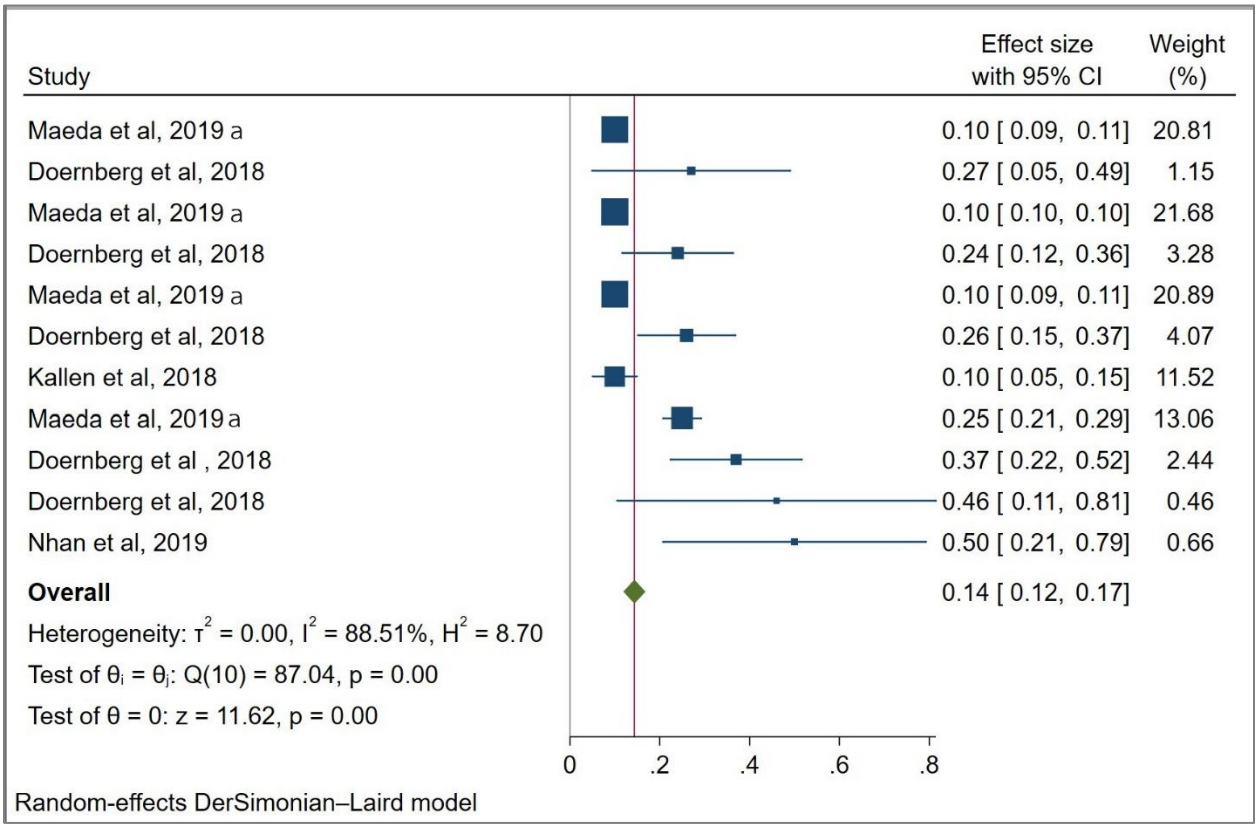

Figure S1 Sensitivity analysis by excluding low quality study. Forest plots for pooling the number of full-time equivalents of ASPs among infectious disease physicians

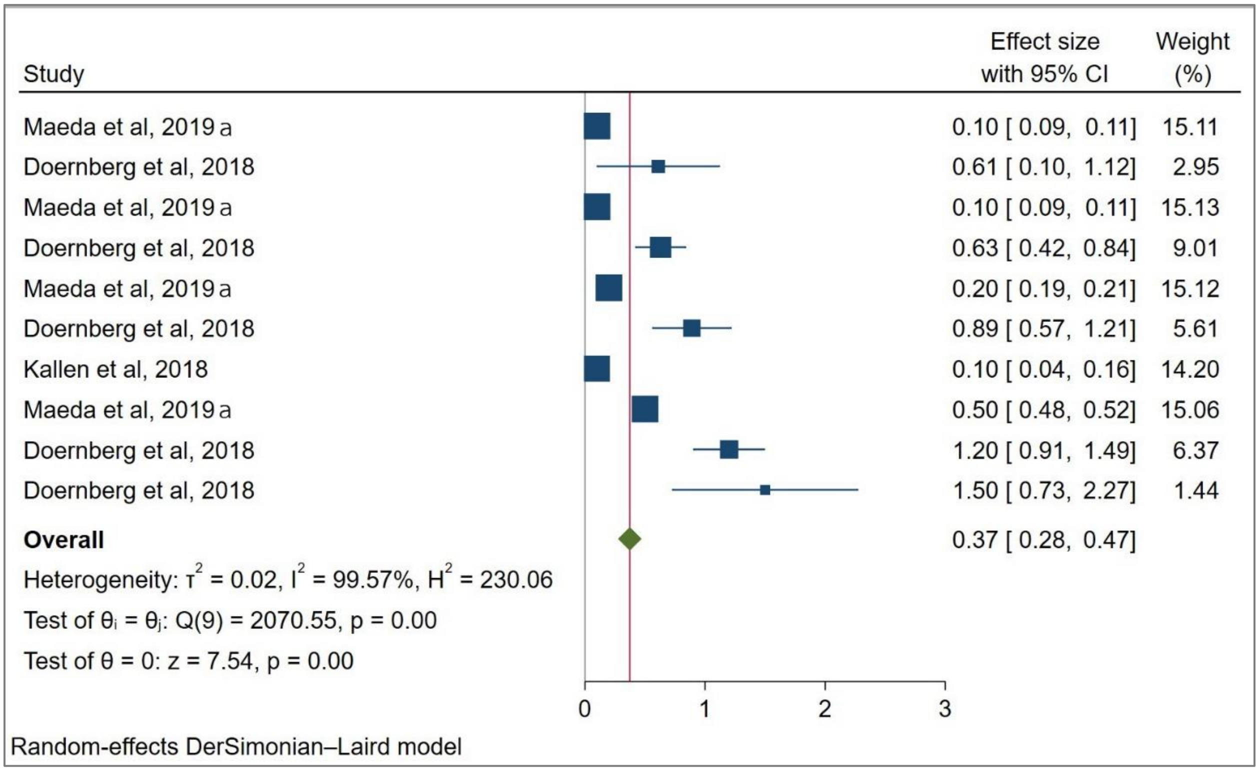

Figure S2 Sensitivity analysis by excluding low quality study. Forest plots for pooling the number of full-time equivalents of ASPs among infectious disease clinical pharmacists

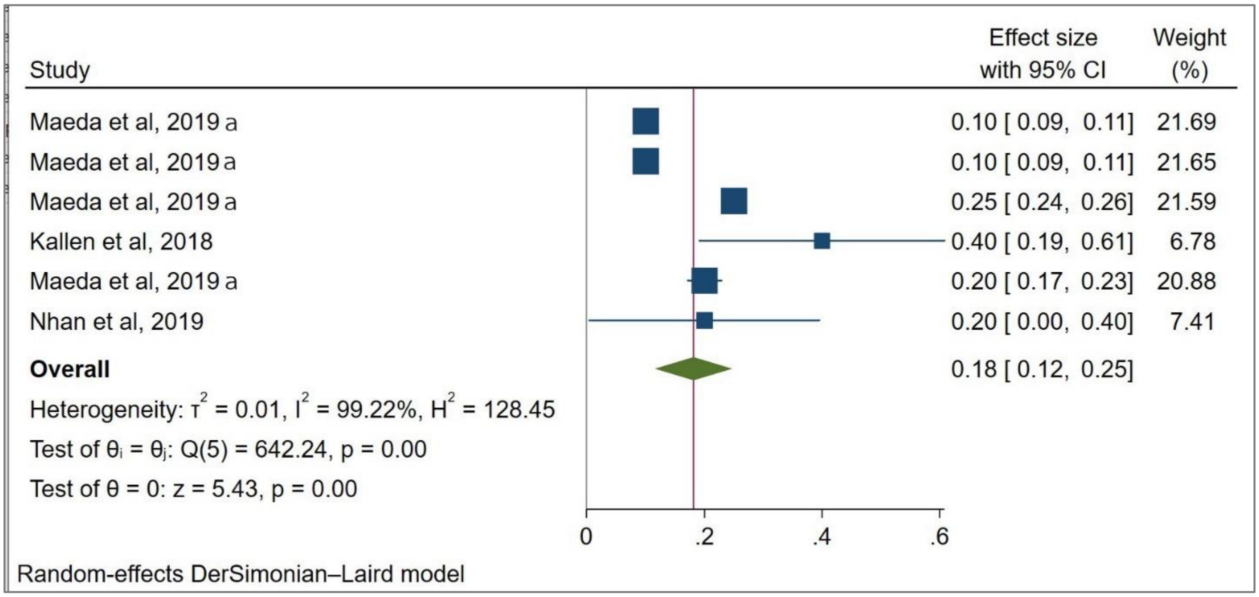

Figure S3 Forest plots for pooling the number of full-time equivalents of ASPs among infection control nurses

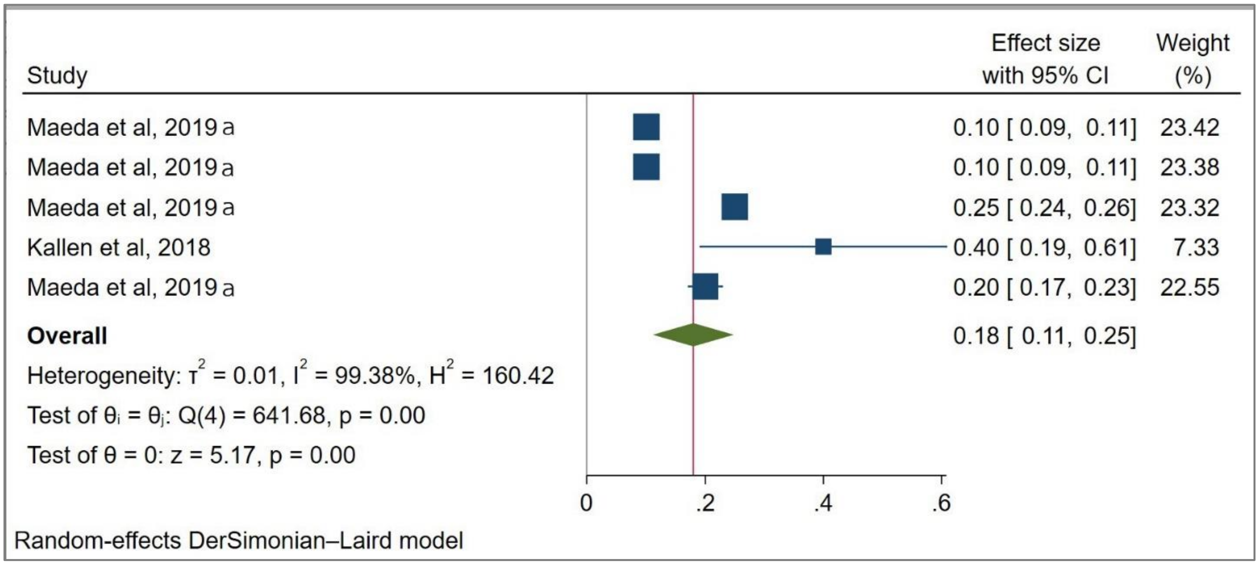

Figure S4 Sensitivity analysis by excluding low quality study. Forest plots for pooling the number of full-time equivalents of ASPs among infection control nurses

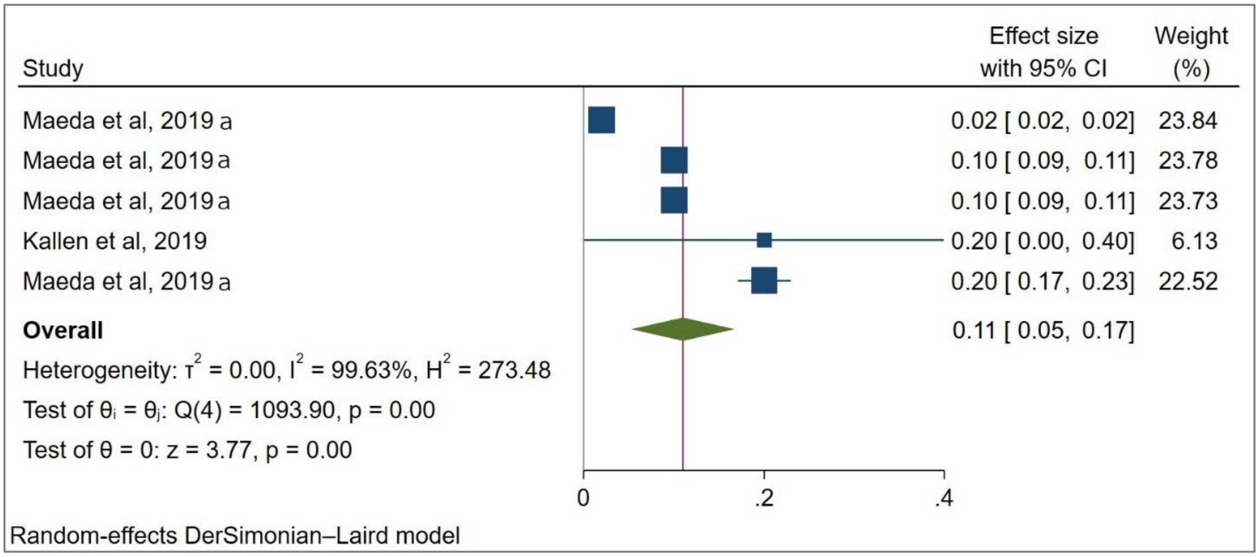

Figure S5 Forest plots for pooling the number of full-time equivalents of ASPs among clinical microbiologists

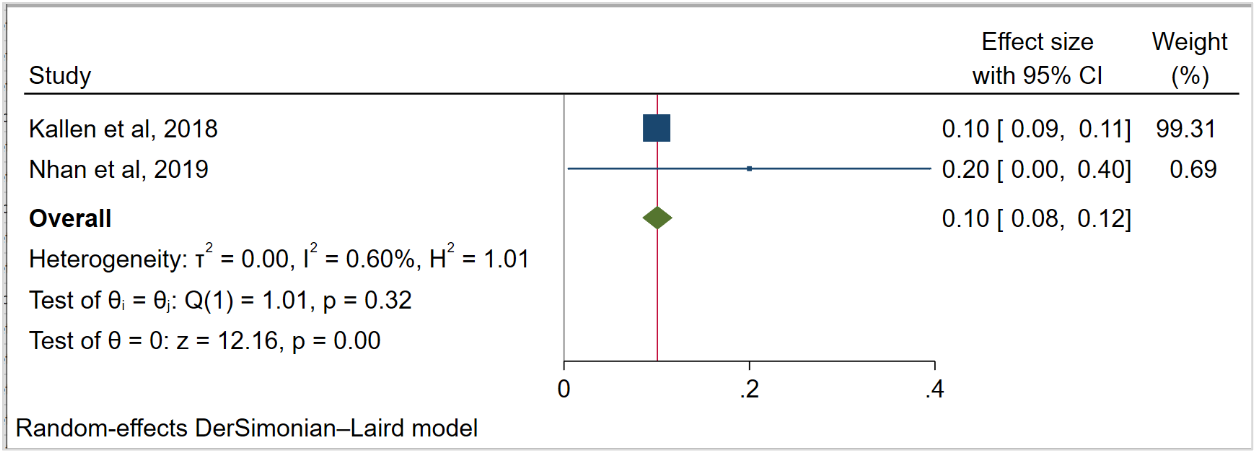

Figure S6 Forest plots for pooling the number of full-time equivalents of ASPs among hospital epidemiologists

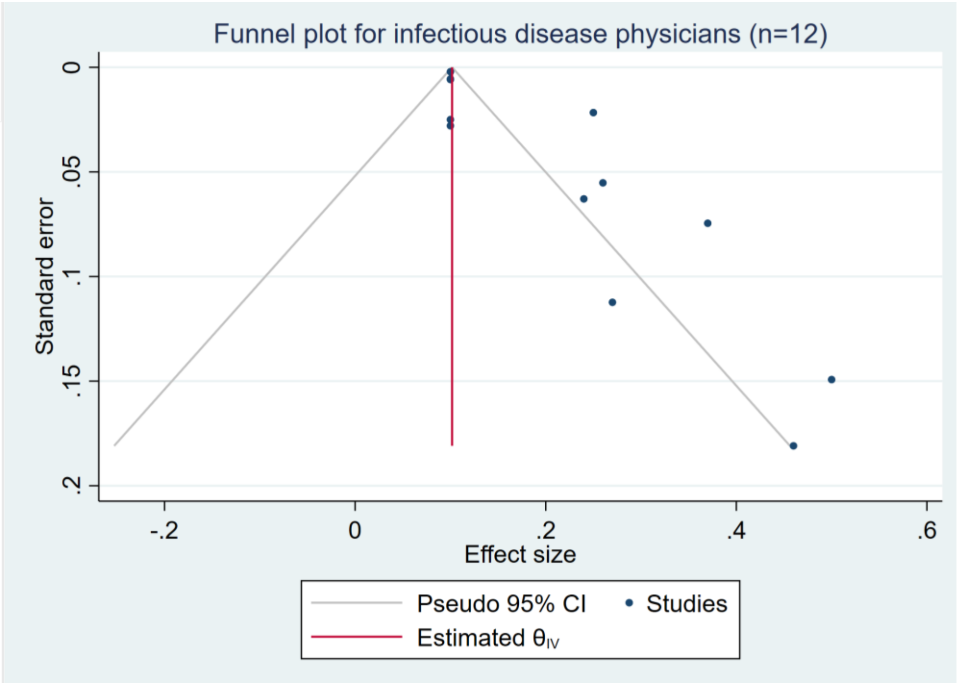

Figure S7 Funnel plots for infectious disease physicians

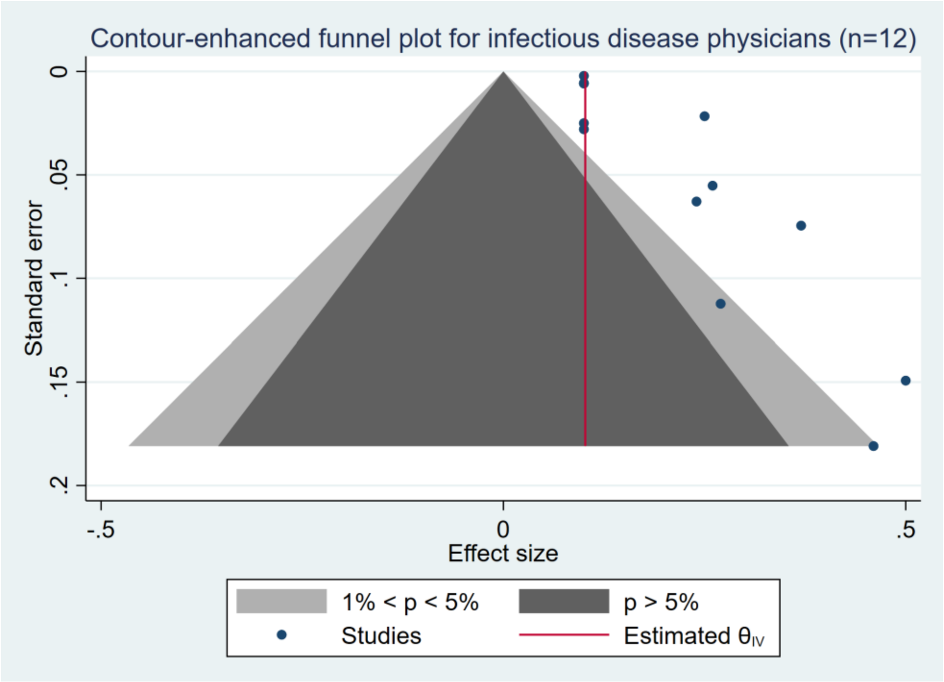

Figure S8 Contour enhanced funnel plots for infectious disease physicians

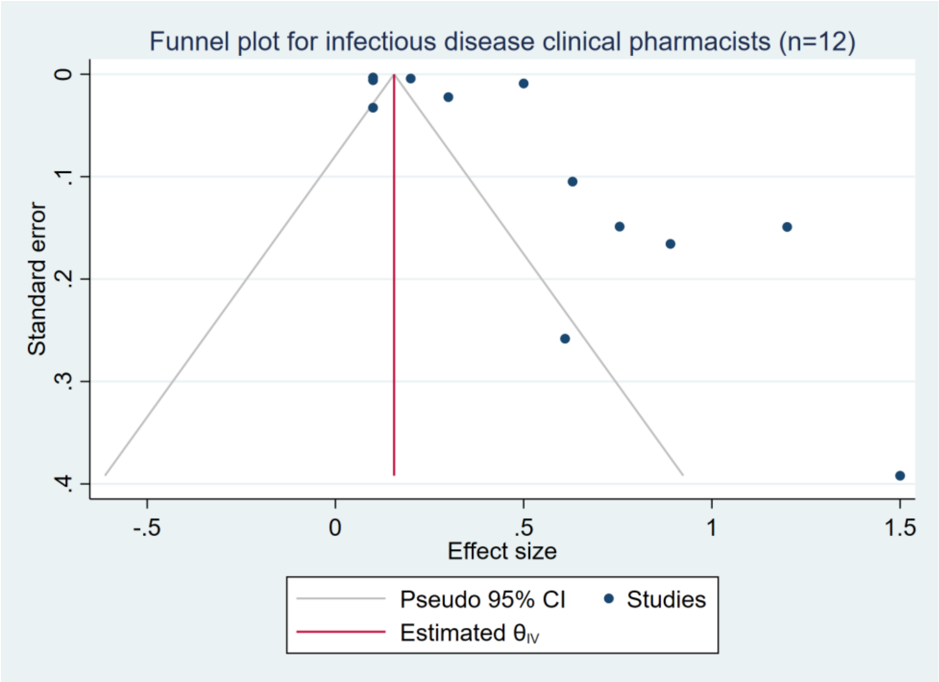

Figure S9 Funnel plots for infectious disease clinical pharmacists

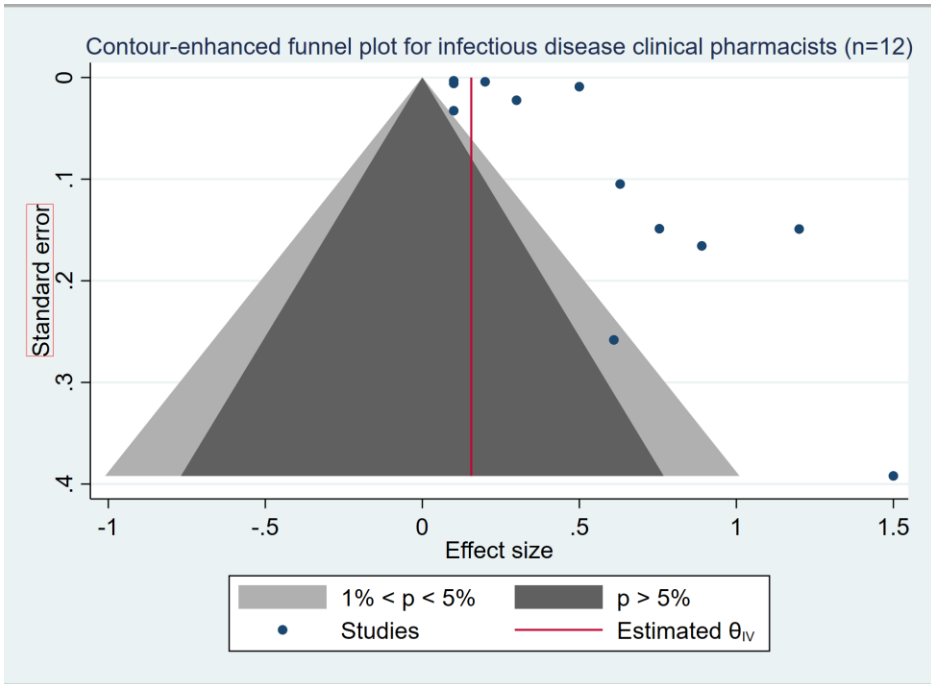

Figure S10 Contour enhanced funnel plots for infectious disease clinical pharmacists

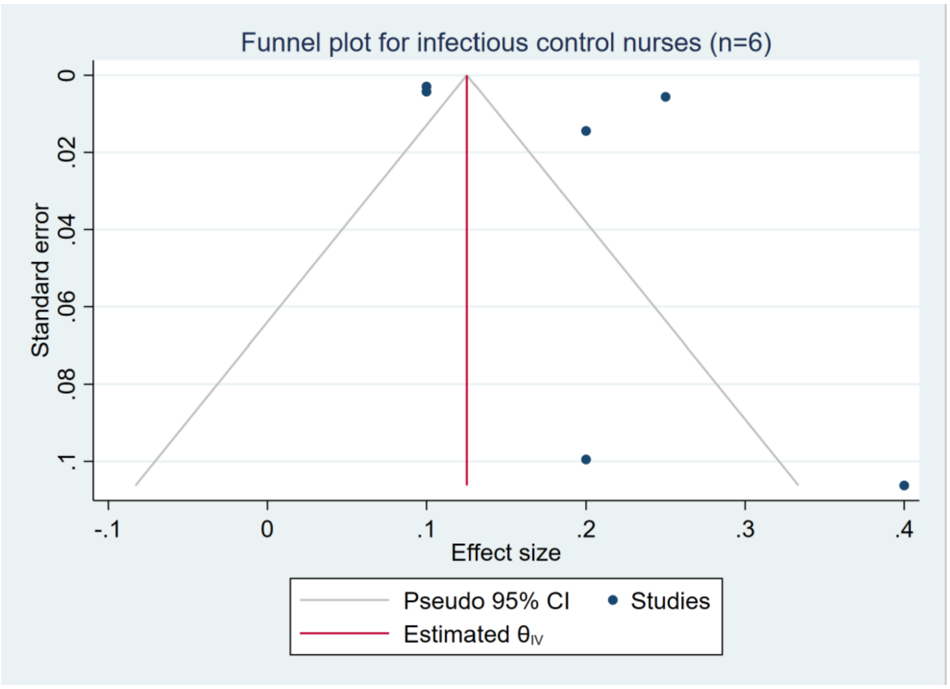

Figure S11 Funnel plots for infection control nurses

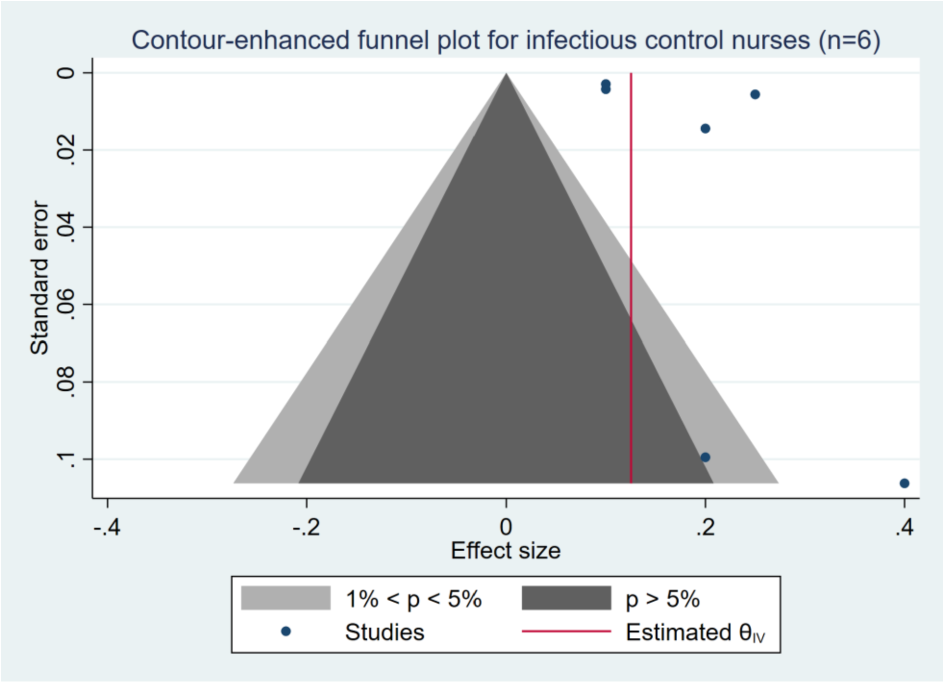

Figure S12 Contour enhanced funnel plots for infection control nurses

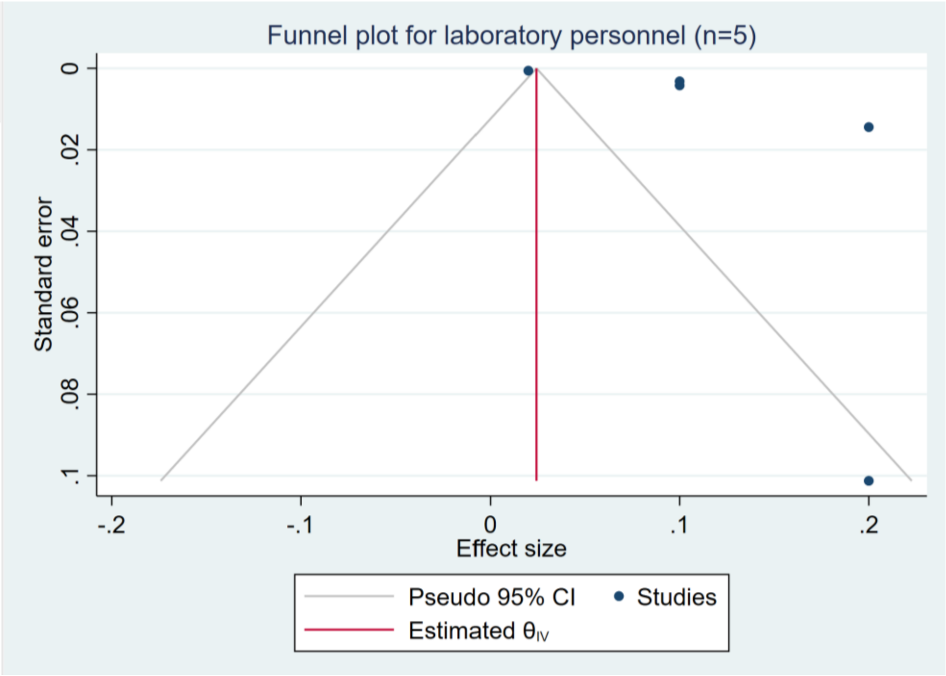

Figure S13 Funnel plots for clinical microbiologists

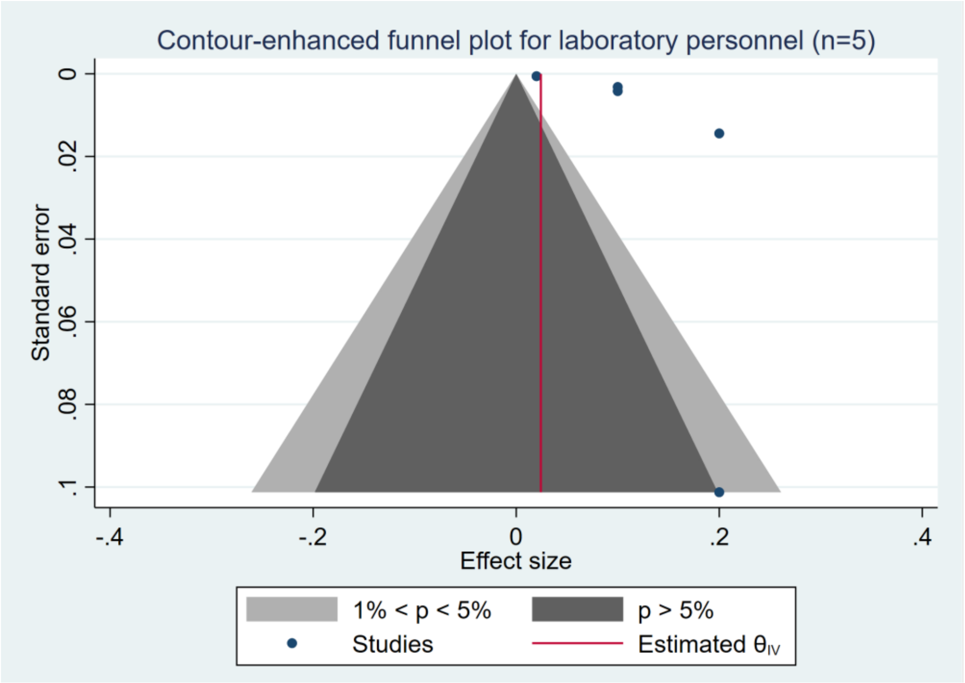

Figure S14 Contour enhanced funnel plots for clinical microbiologists

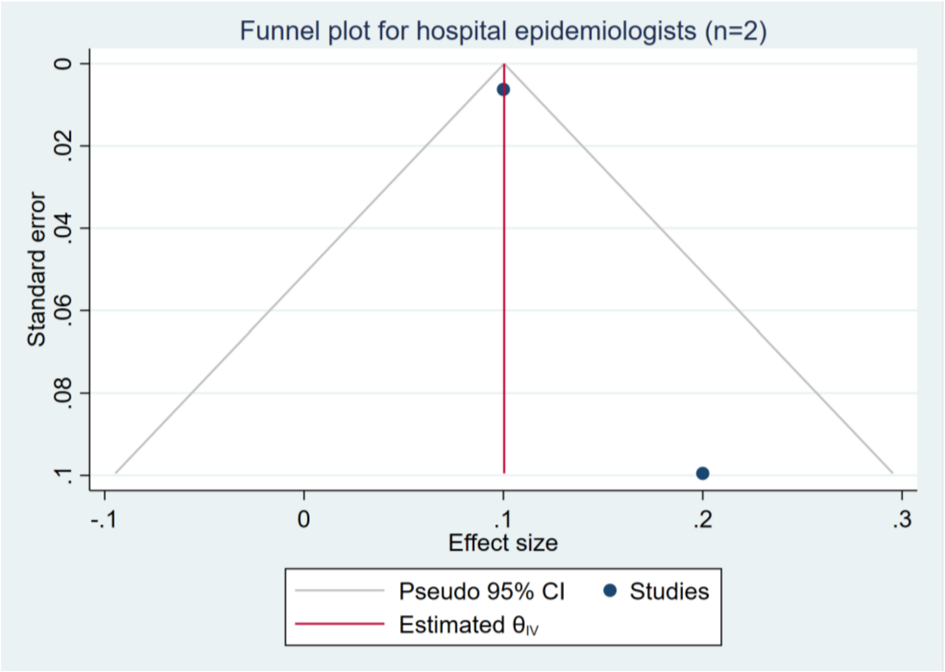

Figure S15 Funnel plots for hospital epidemiologists
